# *In vitro* passage alters virulence, immune activation and proteomic profiles of *Burkholderia pseudomallei*

**DOI:** 10.1038/s41598-020-64914-4

**Published:** 2020-05-20

**Authors:** Taksaon Duangurai, Onrapak Reamtong, Amporn Rungruengkitkun, Varintip Srinon, Usa Boonyuen, Direk Limmathurotsakul, Narisara Chantratita, Pornpan Pumirat

**Affiliations:** 1 Department of Microbiology and Immunology, Faculty of Tropical Medicine, Mahidol University, Bangkok, Thailand; 2 Department of Companion Animal Clinical Sciences, Faculty of Veterinary Medicine, Kasetsart University, Bangkok, Thailand; 3 Department of Molecular Tropical Medicine and Genetics, Faculty of Tropical Medicine, Mahidol University, Bangkok, Thailand; 4 Faculty of Veterinary Science, Veterinary Diagnostic Center, Mahidol University, Nakhon Pathom, Thailand; 5 Mahidol Oxford Tropical Medicine Research Unit, Faculty of Tropical Medicine, Mahidol University, Bangkok, Thailand; 6 Department of Tropical Hygiene, Faculty of Tropical Medicine, Mahidol University, Bangkok, Thailand

**Keywords:** Bacteria, Molecular biology

## Abstract

Serial passage is a problem among many bacterial species, especially those where strains have been stored (banked) for several decades. Prior to banking with an organization such as ATCC, many bacterial strains were passaged for many years, so the characteristics of each strain may be extremely different. This is in addition to any differences in the original host environment. For *Burkholderia pseudomallei*, the number of serial passages should be carefully defined for each experiment because it undergoes adaptation during the course of serial passages. In the present study, we found that passaged *B. pseudomallei* fresh clinical isolates and reference strain in Luria-Bertani broth exhibited increased plaque formation, invasion, intracellular replication, *Galleria mellonella* killing abilities, and cytokine production of host cells. These bacteria also modulated proteomic profiles during *in vitro* passage. We presume that the modulation of protein expression during *in vitro* passage caused changes in virulence and immunogenicity phenotypes. Therefore, we emphasize the need for caution regarding the use of data from passaged *B. pseudomallei*. These findings of phenotypic adaptation during *in vitro* serial passage can help researchers working on *B. pseudomallei* and on other species to better understand disparate findings among strains that have been reported for many years.

## Introduction

*Burkholderia pseudomallei* is a Gram-negative facultative intracellular bacterium and the causative agent of melioidosis, a life threatening disease in humans and animals^1–3^. Melioidosis is an important health problem due to poor understanding of the disease process, frequency of misdiagnosis, and lack of effective vaccination^4,5^. Therefore, *B. pseudomallei* clinical and laboratory isolates have been extensively used to investigate pathogenic mechanisms of bacteria contributing to the disease. Serial passages are routinely performed to maintain an active culture or expand the bacterial number. Serial passages may influence bacterial adaptation through genotypic and phenotypic alterations. In a closely related organism, *Burkholderia mallei*, genome alterations (e.g. gene insertions, deletions, and repetitive sequences) were observed during *in vitro* passage^6^. However, minimal information has been published regarding *B. pseudomallei* adaptation during serial passage. U’ Ren *et al*. examined genomic mutations in *B. pseudomallei* that was serially passaged *in vitro* for 10 days; they found that tandem repeat genome mutations occurred, and suggested that these tandem repeat regions may contribute to generation and maintenance of adaptive genomic variation in *B. pseudomallei*^7^. It was suggested that these tandem repeat regions may play role in generating and maintaining adaptive genomic variation in *B. pseudomallei*^7^. It is likely that *B. pseudomallei* has an unstable genome^7^, which could be beneficial for bacterial adaptation and evolution. In addition, Price *et al*. characterized tandem repeat genome mutations in *B. pseudomallei* isolates from multiple body sites of acute melioidosis patients and found increased numbers and rates of mutations in passaged *B. pseudomallei*^8^. Consistent with those findings, re-sequencing of the laboratory stock of *B. pseudomallei* K96243 (a reference strain commonly used in many laboratories) in 2018 showed genomic alterations compared with the sequence reported in 2004^9^. These studies indicated that *in vitro* passage can alter the genetic of *B. pseudomallei*. In other bacteria, genetic changes can cause phenotypic changes: for example, Mankoski *et al*. demonstrated an increase in *filA* gene expression in *in vivo-*passaged *Helicobacter pylori*, which enhanced bacterial colonization and virulence^10^. Additionally, Somerville *et al*. reported a reduction in aconitase activity of *Staphylococcus aureus* after serial passage for 6 weeks that influence the virulence of passaged *S. aureus*^11^. However, to the best of our knowledge, phenotypic changes in passaged *B. pseudomallei* have not been previously reported.

These prior publications suggest that *in vitro* serial passages of *B. pseudomallei* alter phenotypes of the pathogen, both for reference strain K96243, which is extensively used worldwide among multiple laboratories, and for fresh clinical isolates. Such phenotypic changes of *B. pseudomallei* during serial passage could influence the findings of laboratory experiments, which could lead to misunderstandings regarding the biology of *B. pseudomallei*. We hypothesized that *B. pseudomallei* might alter its protein expression, resulting in phenotypic changes during serial passage. To test this hypothesis, we examined whether serial passage influenced the virulence and immune activation characteristics of fresh clinical isolates of *B. pseudomallei* and reference strain K96243 using plaque formation, invasion efficiency, intracellular replication, and *Galleria mellonella* killing assays. Furthermore, we examined whether serial passage influenced the protein profiles of *B. pseudomallei* fresh clinical isolate and reference strain K96243 during serial passage, using two-dimensional electrophoresis and mass spectrometry. Finally, we examined whether serial passage affected transcription of bacterial genes, using quantitative reverse-transcription polymerase chain reaction (qRT-PCR), in order to validate the proteomic findings.

## Results

### Effect of *B. pseudomallei* serial passage on plaque formation

**Figure 1:**
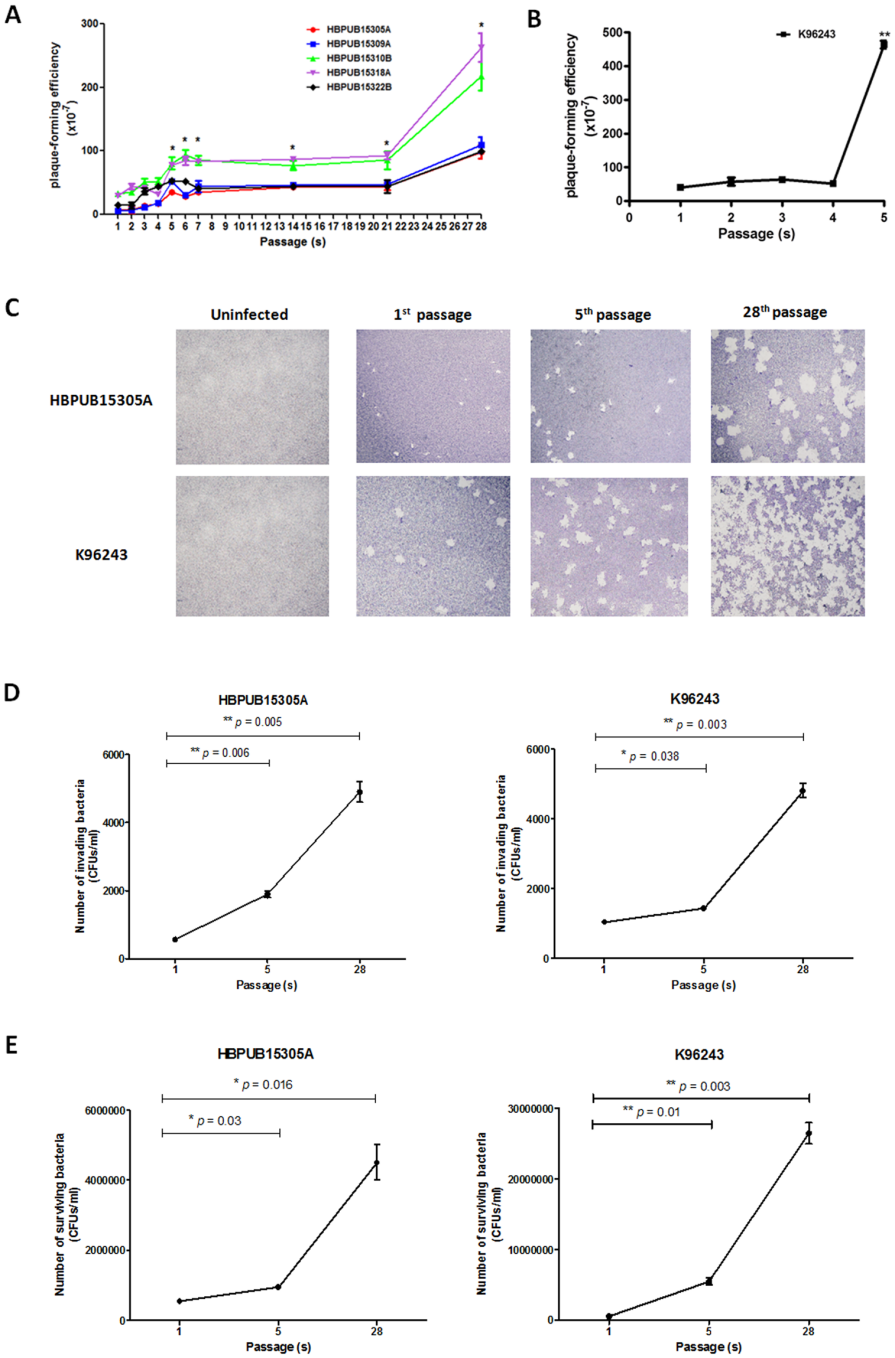
Pathogenic capacities by *B. pseudomallei* fresh clinical isolates and reference strain K96243 that were daily passaged in LB broth for 4 weeks. (**A**) and (**B**) Plaque-forming efficiency of five *B. pseudomallei* fresh clinical isolates and reference strain K96243. (**C**) Photographs of plaques represent images of infected HeLa cell monolayers after infected with passaged *B. pseudomallei* strain HBPUB15305A and reference strain K96243. (**D**) and (**E**) The invasion and intracellular survival of the first, fifth, and 28^th^ passaged *B. pseudomallei* strain HBPUB15305A and reference strain K96243. Asterisks indicate significant differences (*p < 0.05 and **p < 0.01) between groups. Error bars represent standard errors of the means for experiments performed in triplicate.

First, we evaluated the pathogenic capacity of passaged *B. pseudomallei* using the plaque formation assay to determine bacterial abilities regarding invasion, intracellular replication, intracellular survival, and spreading to nearby infected cells. We performed daily passages of *B. pseudomallei* five fresh clinical isolates and reference strain K96243 in LB broth for 28 days (passages 1 to 28). Each passage was assessed for plaque formation. Figures 1A,C show that the plaque-forming efficiencies of the five fresh clinical isolates increased as the passage number increased. In comparison with the first passage, the plaque-forming efficiency was significantly increased after the fifth passage and was highest after the 28^th^ passage. Figures 1B,C show that the plaque-forming efficiency of *B. pseudomallei* reference strain K96243 was significantly increased after the fifth passage. Unexpectedly, the plaque-forming efficiencies of *B. pseudomallei* reference strain K96243 after subsequent passages could not be calculated because of the lysis of large numbers of infected HeLa cells. Means and standard deviations (SDs) of the plaque forming-efficiencies of *B. pseudomallei* reference strain K96243 were 39.0 ± 2.7 (first passage), 59.7 ± 9.3 (second passage), 67.0 ± 2.7 (third passage), 64.3 ± 21.6 (fourth passage), and 447.0 ± 31.1 (fifth passage), while those for fresh clinical isolates were 18.2 ± 12.1 (first passage), 21.7 ± 16.1 (second passage), 31.2 ± 16.4 (third passage), 32.6 ± 14.7 (fourth passage), and 59.7 ± 18.5 (fifth passage). Notably, the plaque-forming efficiency of *B. pseudomallei* reference strain K96243 was higher than the efficiencies of fresh clinical isolates in all passages. Figure 1C demonstrates the images of plaque formation of HeLa cells infected with *B. pseudomallei* prepared after one, five, and 28 passages, in comparison with uninfected cells. These images show that plaque formation increased in association with an increased passage number. This finding indicated that *in vitro* serial passage affected *B. pseudomallei* pathogenesis.

### Ability of *in vitro* passaged *B. pseudomallei* to invade and intracellularly replicate in HeLa cells

Next, we selected *B. pseudomallei* strains prepared after one, five, and 28 passages to test their abilities to invade and replicate inside HeLa cells. The invasion abilities of the first, fifth, and 28^th^ passages of *B. pseudomallei* fresh clinical isolate and reference strain K96243 were compared. Because there were no differences in the growth kinetics of all fresh clinical isolates (data not shown), we selected fresh clinical strain HBPUB15305A as a representative of fresh clinical isolates. Means and SDs of invasion of the *B. pseudomallei* strain HBPUB15305A prepared after one, five, and 28 passages were 570 ± 14 CFUs/ml, 1900 ± 141 CFUs/ml, and 4900 ± 424 CFUs/ml, respectively. This indicated that the bacteria from the fifth and 28^th^ passages were more invasive than those from the first passage. Furthermore, the invasion ability of bacteria prepared from the 28^th^ passage of *B. pseudomallei* strain HBPUB15305A was significantly increased, compared with the invasion ability of bacteria prepared from the fifth passage of this isolate (*p* = 0.007) (Fig. 1D). Moreover, means and SDs of invasion of *B. pseudomallei* reference strain K96243 prepared after one, five, and 28 passages were 1050 ± 70 CFUs/ml, 1440 ± 84 CFUs/ml, and 4800 ± 282 CFUs/ml, respectively. The results of *B. pseudomallei* reference strain K96243 were in good agreement with those of the fresh clinical isolate; bacteria from the fifth and 28^th^ passages were significantly more invasive than those from the first passage (Fig. 1D).

At 12 h post-infection, intracellular replications of passaged *B. pseudomallei* were detected in HeLa cells. Means and SDs of intracellular survival of *B. pseudomallei* strain HBPUB15305A prepared after one, five, and 28 passages were 55.0 ± 7.1 × 10^4^ CFUs/ml, 95.0 ± 7.1 × 10^4^ CFUs/ml, and 450.0 ± 7.1 × 10^4^ CFUs/ml, respectively. The numbers of intracellular bacteria prepared from the fifth and 28^th^ passages of *B. pseudomallei* strain HBPUB15305A were significantly higher than those of bacteria prepared from the first passage of this isolate (*p* = 0.006 and 0.008, respectively) (Fig. 1E). In addition, means and SDs of intracellular survival of *B. pseudomallei* reference strain K96243 prepared after one, five, and 28 passages were 57.5 ± 3.5 × 10^4^ CFUs/ml, 550.0 ± 70.7 × 10^4^ CFUs/ml, and 2650.0 ± 212.0 × 10^4^ CFUs/ml, respectively. Likewise, the numbers of intracellular bacteria prepared from the fifth and 28^th^ passages of *B. pseudomallei* reference strain K96243 were significantly higher than those of bacteria prepared from the first passage of this strain (*p* = 0.004 and 0.002, respectively) (Fig. 1E). Our results demonstrated that *in vitro* serial passage affected *B. pseudomallei* pathogenesis in terms of invasion and intracellular survival.

### Pathogenicity of passaged *B. pseudomallei* toward *G. mellonella*

**Figure 2:**
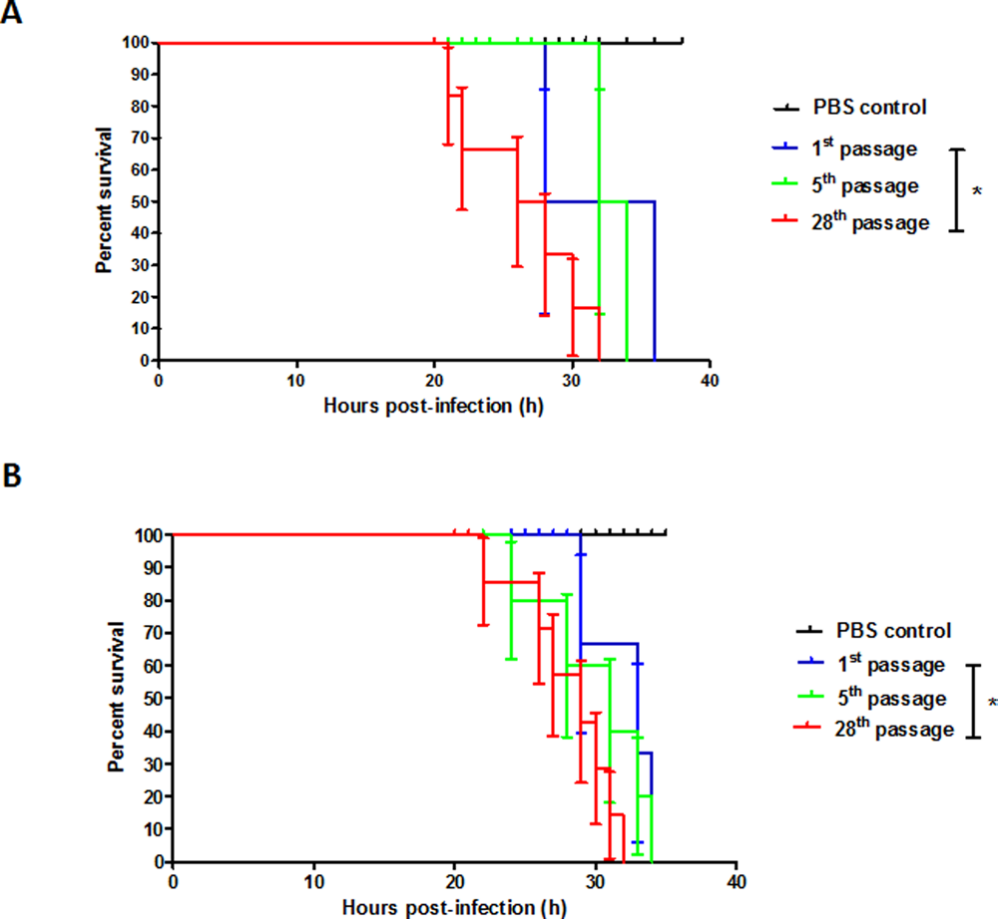
Survival of *G. mellonella* larvae infected with passaged *B. pseudomallei* was monitored over 40 h. (**A**) *G. mellonella* larvae were injected with the first, fifth, and 28^th^ passaged *B. pseudomallei* strain HBPUB15305A (10^2^ CFUs). (**B**) *G. mellonella* larvae were injected with the first, fifth, and 28^th^ passaged *B. pseudomallei* reference strain K96243 (10^2^ CFUs). Each data set is a representative of a single trial with % survival of infected larvae. Control larvae (PBS injected larvae) did not die in any given trial. Asterisks indicate significant differences (*p* < 0.05).

On the basis of *in vitro* plaque formation, invasion, and intracellular replication assays, we hypothesized that passaged *B. pseudomallei* may also exhibited increased virulence in an animal model. The *G. mellonella* killing assay was performed to test this hypothesis. *G. mellonella* larvae were infected with *B. pseudomallei* prepared after one, five, and 28 passages. PBS was used as a control. Larvae survival was monitored over time following the injection of bacterial suspension (Fig. 2). For *B. pseudomallei* strain HBPUB15305A, larvae infected with bacteria prepared from the 28^th^ passage died more rapidly than larvae infected with bacteria prepared from the first passage (*p* = 0.028) (Fig. 2A). At 32 h post-infection, larvae infected with bacteria prepared from the 28^th^ passage of *B. pseudomallei* strain HBPUB15305A exhibited to 100% mortality. In contrast, 100% mortality was observed at 34 and 36 h post-infection for larvae infected with bacteria prepared from the fifth and first passages of *B. pseudomallei* strain HBPUB15305A, respectively. Likewise, larvae infected with bacteria prepared from the 28^th^ passage of *B. pseudomallei* reference strain K96243 died more rapidly than larvae infected with bacteria prepared from the first passage (*p* = 0.045) (Fig. 2B). They exhibited 100% mortality at 32 h post-infection. However, larvae infected with bacteria prepared from the fifth and first passages of *B. pseudomallei* reference strain K96243 showed 100% mortality at 34 h post-infection. No mortality was observed in control PBS-injected larvae. These findings show that *in vitro* serial passage of *B. pseudomallei* enhanced bacterial virulence, leading to increased mortality in *G. mellonella*. These *in vivo* results are consistent with our *in vitro* findings, indicating enhanced virulence capacity in passaged *B. pseudomallei*.

### Proteomic alteration in *in vitro* passaged *B. pseudomallei*

**Figure 3:**
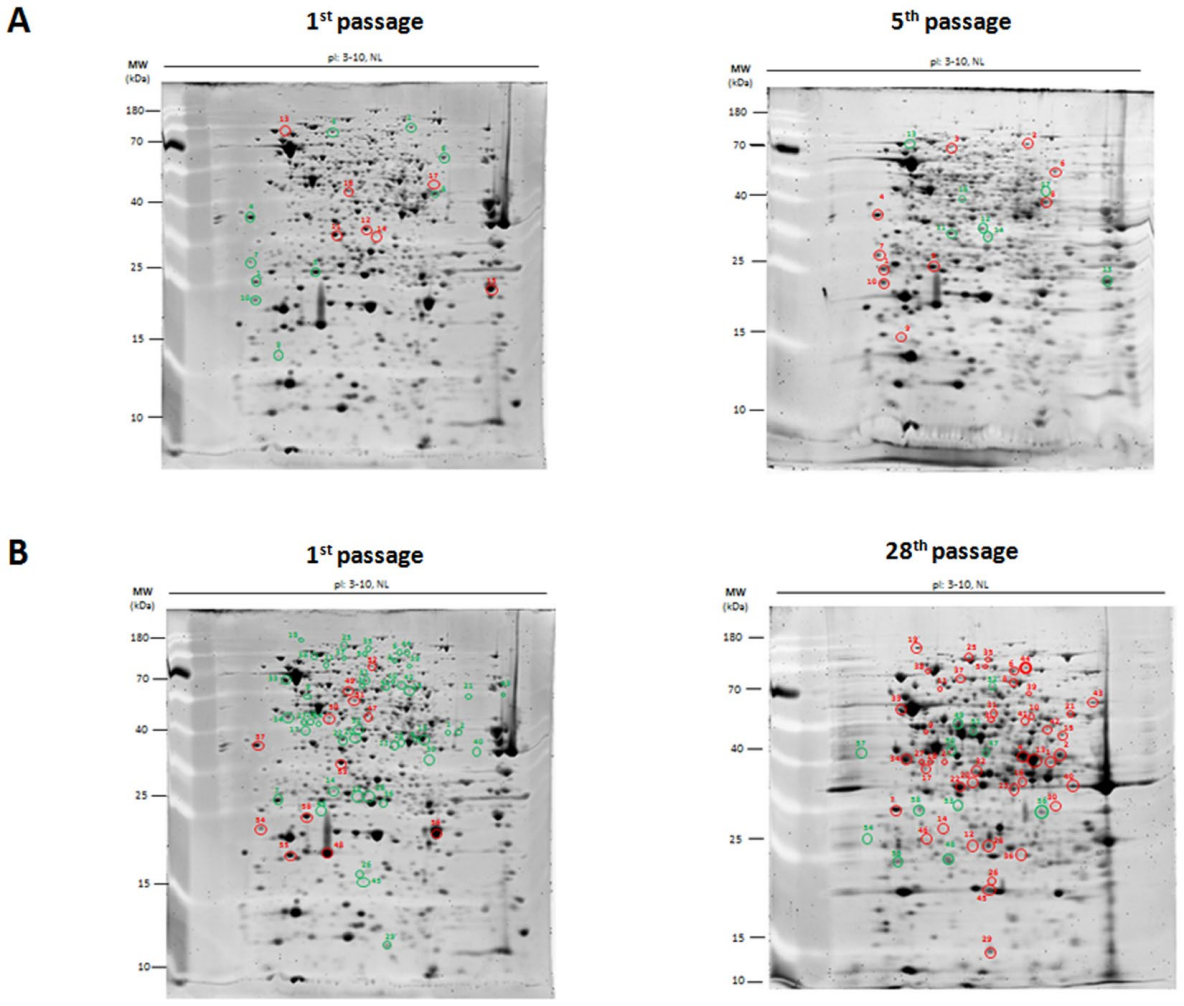
The comparison of proteomic profiles of passaged *B. pseudomallei* strain HBPUB15305A. (**A**) and (**B**) The comparison of proteomic profiles of the fifth and 28^th^ passaged *B. pseudomallei* strain HBPUB15305A to the first passage. Red circles indicate up-regulated proteins. Green circles indicate down-regulated proteins. Additional information is provided in Supplementary Tables S1 and S2.

To gain more information regarding the mechanism involved in increased pathogenicity during serial passage, we performed a comparative proteomic analysis of the selected *B. pseudomallei* strain HBPUB15305A, using bacteria prepared from the first, fifth, and 28^th^ passages. Figures 3A,B show the comparative proteome profiles of bacteria prepared from the first versus fifth passages and bacteria prepared from the first versus 28^th^ passages of *B. pseudomallei* strain HBPUB15305A, respectively. In comparison with bacteria prepared from the first passage, 17 of 670 protein spots were identified as altered proteins in bacteria prepared from the fifth passage. Among these proteins, 10 protein spots were significantly upregulated, while seven protein spots were downregulated in bacteria prepared from the fifth passage (Fig. 3A and Supplementary Figure S1). In contrast, 58 of 845 protein spots were altered in bacteria prepared from the 28^th^ passage, compared with bacteria prepared from the first passage. Among these proteins, 46 were upregulated, while 12 were downregulated (Fig. 3B and Supplementary Figure S1).

**Figure 4:**
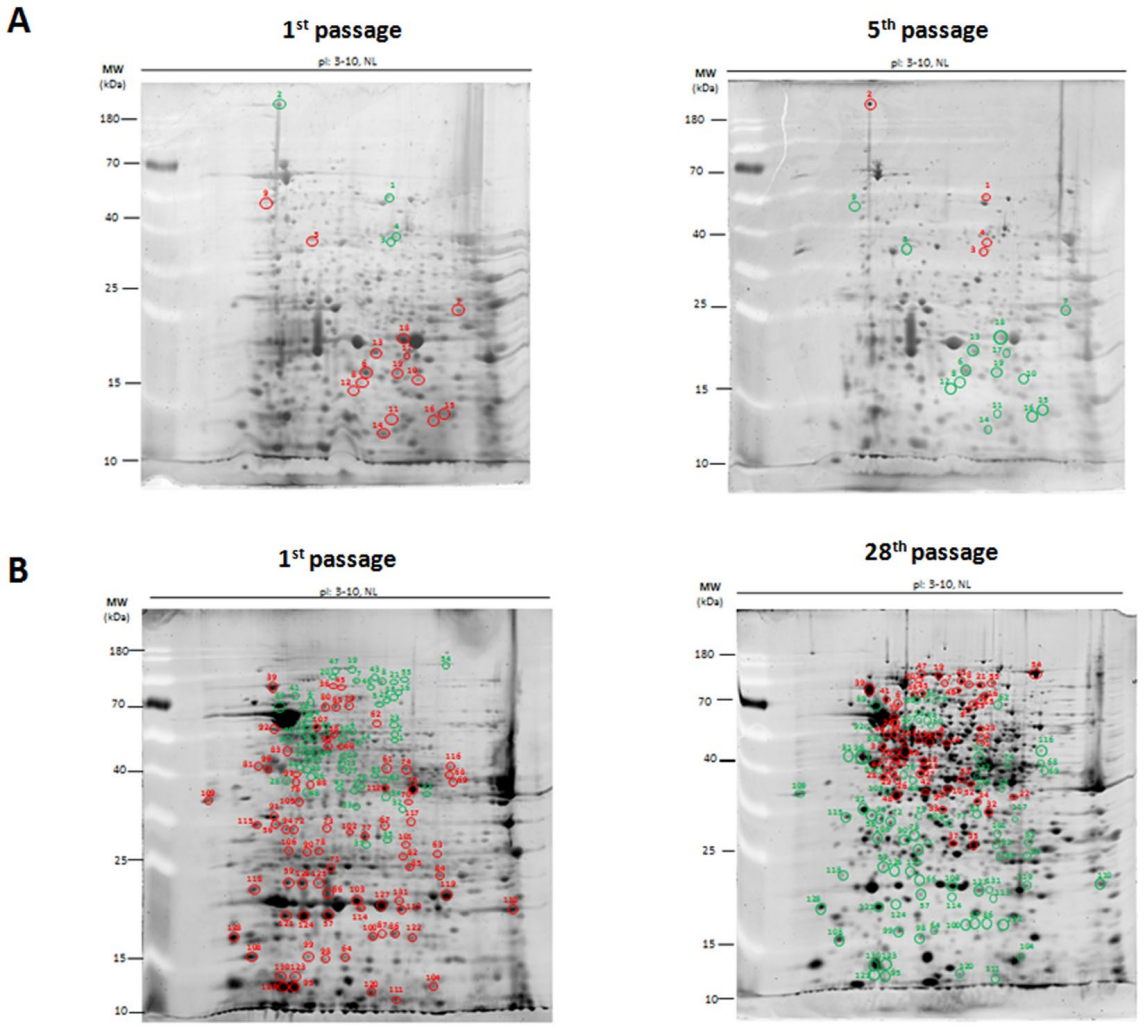
The comparison of proteomic profiles of passaged *B. pseudomallei* reference strain K96243. (**A**) and (**B**) The comparison of proteomic profiles of the fifth and 28^th^ passaged *B. pseudomallei* reference strain K96243 to the first passage. Red circles indicate up-regulated proteins. Green circles indicate down-regulated proteins. Additional information is provided in Supplementary Tables S3 and S4.

Figure 4 show the comparative proteome profiles of bacteria prepared from the first versus fifth passages and bacteria prepared from the first versus 28^th^ passages of *B. pseudomallei* reference strain K96243, respectively. In comparison with bacteria prepared from the first passage, 19 of 786 protein spots were identified as altered proteins in bacteria prepared from the fifth passage. Among these proteins, 4 protein spots were significantly upregulated, while 15 protein spots were downregulated in bacteria prepared from the fifth passage (Fig. 4A and Supplementary Figure S2). In contrast, 109 of 845 protein spots were altered in bacteria prepared from the 28^th^ passage, compared with bacteria prepared from the first passage. Among these proteins, 55 were upregulated, while 54 were downregulated (Fig. 4B and Supplementary Figure S2).

**Figure 5:**
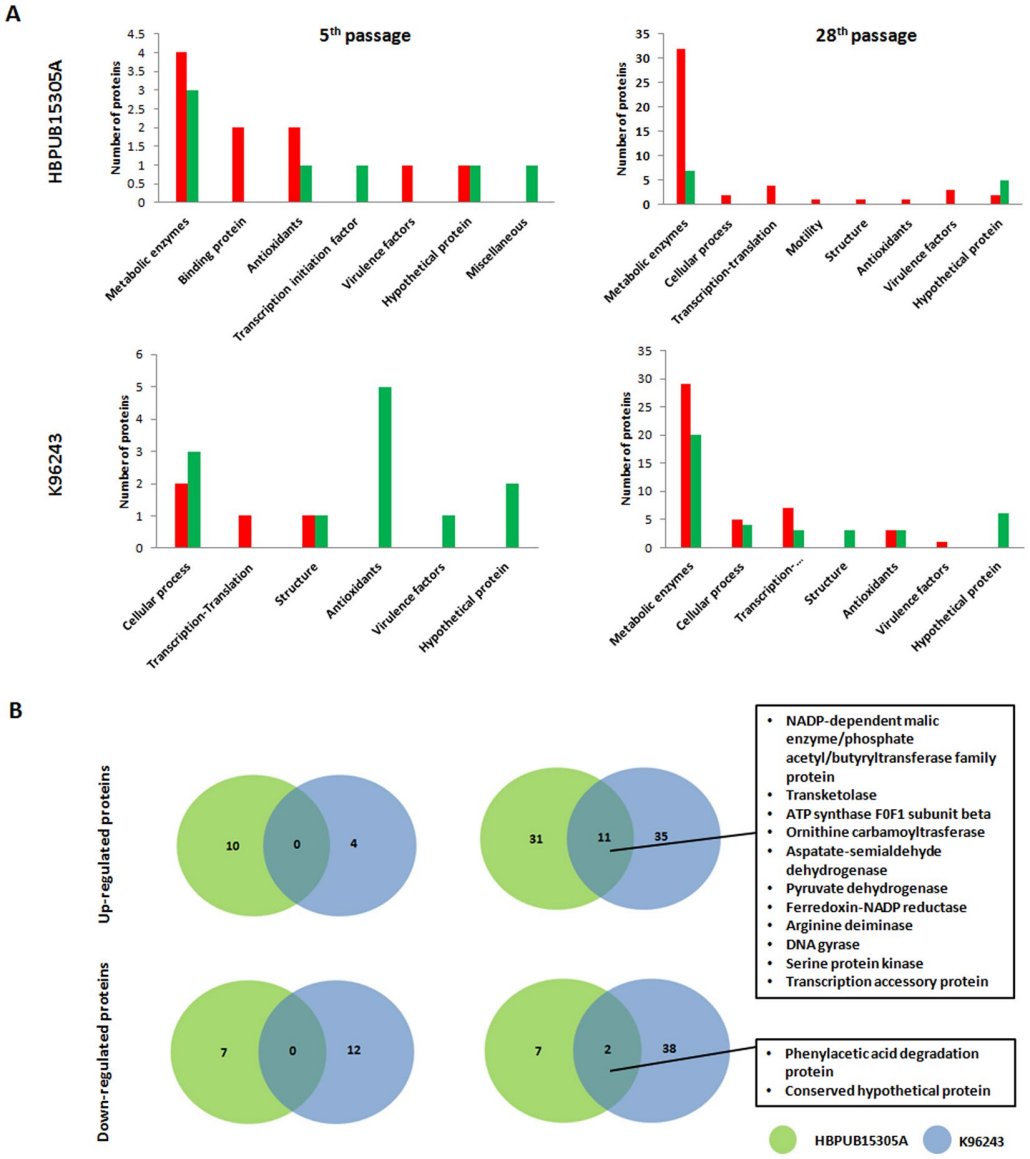
Protein alteration of passaged *B. pseudomallei*. (**A**) The proteins were categorized based on biological function by Uniport database. Red bar represents increased number of proteins, and green bar represents decreased number of proteins. (**B**) Venn diagrams show the number of expressed proteins identified in passaged *B. pseudomallei* strain HBPUB15305A (green) and reference strain K96243 (blue). These diagrams show the distribution of the number of up-regulated and down-regulated proteins in the fifth and 28^th^ passaged *B. pseudomallei* strain HBPUB15305A and reference strain K96243. The identified proteins of overlapping sections are listed on the right.

Next, the altered protein spots from bacteria prepared from the first, fifth, and 28^th^ passages of *B. pseudomallei* strain HBPUB15305A and reference strain K96243 were subsequently identified using liquid chromatography–tandem mass spectrometry (LC-MS/MS) (Supplementary Tables S1–S4). For *B. pseudomallei* strain HBPUB15305A, the 10 upregulated proteins in bacteria prepared from the fifth passage were classified into five different functional groups: metabolic pathways, binding proteins, antioxidants, virulence factors, and hypothetical proteins; the 7 downregulated proteins in bacteria prepared from the fifth passage were classified as proteins involved in metabolic pathways, antioxidants, transcription initiation factors, hypothetical proteins, and miscellaneous (Supplementary Tables S1 and Fig. 5A). Furthermore, the 46 upregulated proteins in bacteria prepared from the 28^th^ passage of *B. pseudomallei* strain HBPUB15305A were classified as metabolic enzymes, proteins involved in transcription-translation processes, virulence factors, bacterial cellular process, antioxidants, motility proteins, membrane structural proteins, and hypothetical proteins (Supplementary Tables S2 and Fig. 5A); the 12 downregulated proteins in bacteria prepared from the 28^th^ passage were classified as proteins involved in metabolic pathways and hypothetical proteins. Several upregulated proteins in *B. pseudomallei* strain HBPUB15305A were involved in bacterial virulence; these included arginine deiminase, ferritin, flagellar biosynthesis protein, flavohemoprotein, porin, oxidoreductase, serine protease, serine protein kinase, and transcription accessory protein (Tex).

For *B. pseudomallei* reference strain K96243, the 4 upregulated proteins in bacteria prepared from the fifth passage were classified as proteins involved in cellular processes, proteins involved in transcription-translation processes, and proteins involved in structural roles; the 12 downregulated proteins in bacteria prepared from the fifth passage were classified as antioxidant proteins, proteins involved in cellular processes, structural proteins, virulence factors, and hypothetical proteins (Supplementary Tables S3 and Fig. 5A). Furthermore, the 45 upregulated proteins in bacteria prepared from the 28^th^ passage of *B. pseudomallei* reference strain K96243 were classified as proteins involved in metabolic pathways, proteins involved in cellular processes, proteins involved in transcription-translation processes, antioxidants, and virulence factors (Supplementary Tables S4 and Fig. 5A); the 39 downregulated proteins in bacteria prepared from the 28^th^ passage were identified as proteins involved in metabolic pathways, proteins involved in cellular processes, proteins involved in transcription-translation processes, structural proteins, antioxidants, and hypothetical proteins. Several upregulated proteins in the passaged reference strain K96243 were involved in bacterial virulence; these included arginine deiminase, chaperone GroEL, chaperone GroL, elongation factor TU, porin, oxidoreductase, serine protein kinase, and Tex.

A comparison between *B. pseudomallei* strain HBPUB15305A and reference strain K96243 demonstrated that, in bacteria prepared from the 28^th^ passage, 11 proteins were upregulated in both strains, all of which were involved in metabolic pathways and virulence (Fig. 5B): ATP synthase F0F1 subunit beta, arginine deiminase, aspartate-semialdehyde dehydrogenase, DNA gyrase, NADP-dependent malic enzyme/phosphate acetyl/butyryl transferase family protein, ferredoxin-NADP reductase, ornithine carbamoyltransferase, pyruvate dehydrogenase, serine protein kinase, Tex, and transketolase. In addition, two proteins (phenylacetic acid degradation and hypothetical protein) were downregulated in bacteria prepared from both *B. pseudomallei* strain HBPUB15305A and reference strain K96243 (Fig. 5B). However, up- and downregulated proteins were not similar in bacteria prepared from the fifth passages of *B. pseudomallei* strain HBPUB15305A and reference strain K96243 (Fig. 5B). Furthermore, the comparative proteome profiles of bacteria prepared from the fifth and 28^th^ passages of *B. pseudomallei* strain HBPUB15305A revealed that 38 protein spots were upregulated, of which 34 were identical. These upregulated proteins were mostly associated with *B. pseudomallei* metabolic processes.

**Figure 6:**
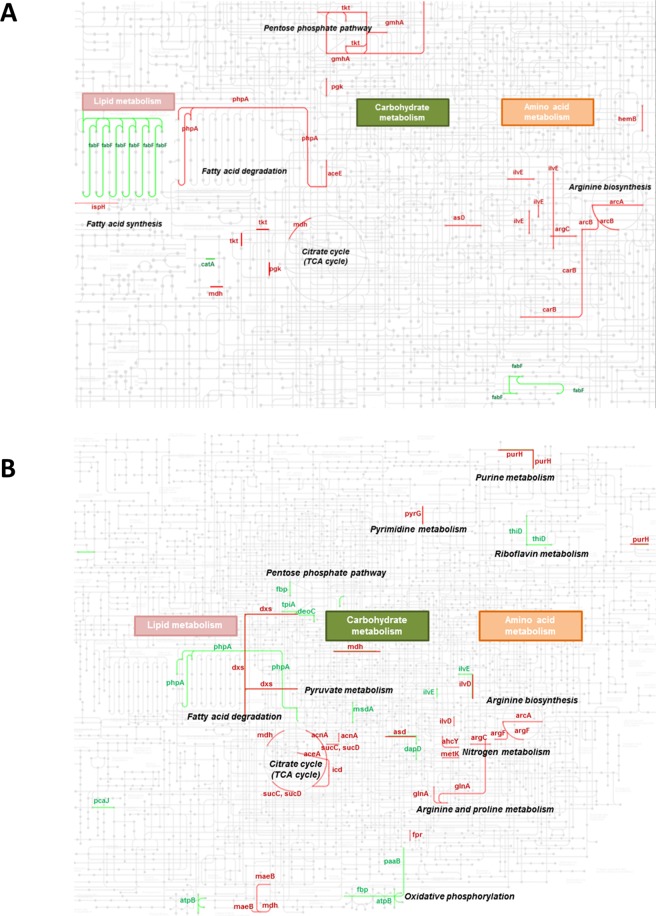
KEGG pathway analysis. (**A**) and (**B**) KEGG pathway result of *B. pseudomallei* strain HBPUB15305A and reference strain K96243. Up- and down-regulated proteins are labeled in red and green, respectively.

To further analyze the functional roles of proteins that were differentially expressed between bacteria prepared from the 28^th^ passages of *B. pseudomallei* strain HBPUB15305A and reference strain K96243, we performed KEGG pathway analysis (Figs. 6A,B). We found that the majority of upregulated proteins in both strains were involved metabolic pathways (Figs. 6A,B), including pentose phosphate, lipoic acid metabolism, fatty acid degradation, arginine biosynthesis, and amino acid metabolism pathways. In contrast, proteins involved in fatty acid biosynthesis were downregulated in both *B. pseudomallei* strain HBPUB15305A and reference strain K96243 (Figs. 6A,B). However, we did not generate a KEGG map for altered proteins in bacteria prepared from the fifth passages of *B. pseudomallei* strain HBPUB15305A and reference strain K96243 because only a few altered proteins were identified in KEGG analysis. These proteomic findings suggest that *B. pseudomallei* bacteria can adjust their metabolic pathways during serial passage.

### Transcriptomic expression of passaged *B. pseudomallei*

**Figure 7:**
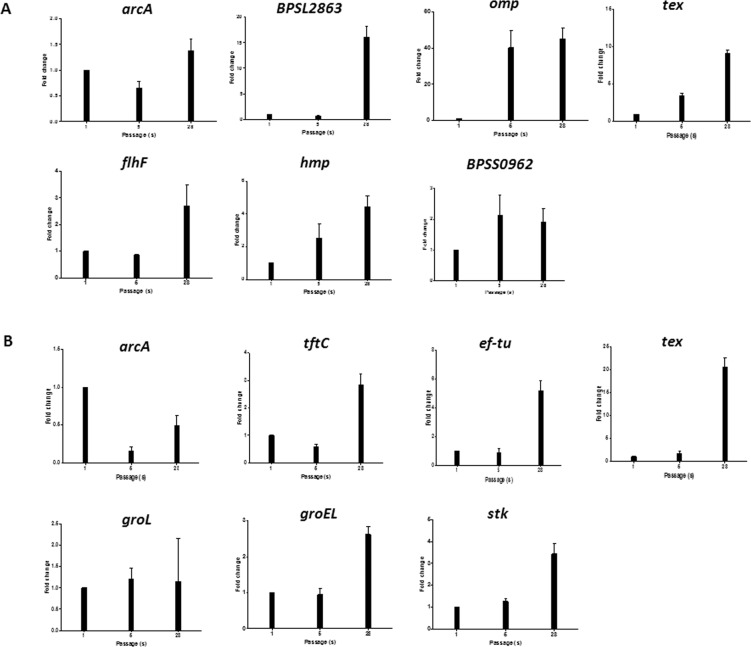
Transcriptomic expression of passaged *B. pseudomallei* strain HBPUB15305A and reference strain K96243. (**A**) The fold change in gene expression of the *arcA, flhF, hmp, omp, tex, BPSL2863*, and *BPSS0962* of the first, fifth and 28^th^ passaged *B. pseudomallei* strain HBPUB15305A. (**B**) The fold change in gene expression of the *arcA*, *ef-tu*, *groEL*, *groL*, *stk*, *tex*, and *tftC* of the first, fifth, and 28^th^ *B. pseudomallei* reference strain K96243. 23S RNA was used as a reference for calculation of relative expression levels. The normalised expression levels were calculated by using 2^−ΔΔCT^ method. Data represent the mean ± standard deviation.

In this study, we performed transcriptomic expression analysis by qRT-PCR to verify proteomic changes in passaged *B. pseudomallei*, using both *B. pseudomallei* strain HBPUB15305A and reference strain K96243. Twelve genes formerly reported in bacterial virulence were selected; these included *arcA, ef-tu, flhF, groEL, groL, hmp, omp, stk, tex, tftC, BPSL2863*, and *BPSS0962*. qRT-PCR results showed that the expression levels of *arcA*, *flhF*, *hmp*, *omp*, *tex*, *BPSL2863*, and *BPSS0962* genes were noticeably increased in bacteria prepared from the 28^th^ passage of *B. pseudomallei* strain HBPUB15305A, compared with bacteria prepared from the first passage (Fig. 7A). Additionally, the expression levels of *hmp*, *omp*, *tex*, and *BPSS0962* genes were also increased in bacteria prepared from the fifth passage of *B. pseudomallei* strain HBPUB15305A, compared with bacteria prepared from the first passage (Fig. 7A). Compared with bacteria prepared from the first passage, bacteria prepared from the 28^th^ passage of *B. pseudomallei* reference strain K96243 showed increased expression levels of *arcA, ef-tu, groEL, groL, stk, tex*, and *tftC* genes (Fig. 7B). Thus, the mRNA expression results were consistent with the proteomic findings.

### Effect of serial passage of *B. pseudomallei* strain HBPUB15305A on pro-inflammatory cytokine production by infected host cells

**Figure 8:**
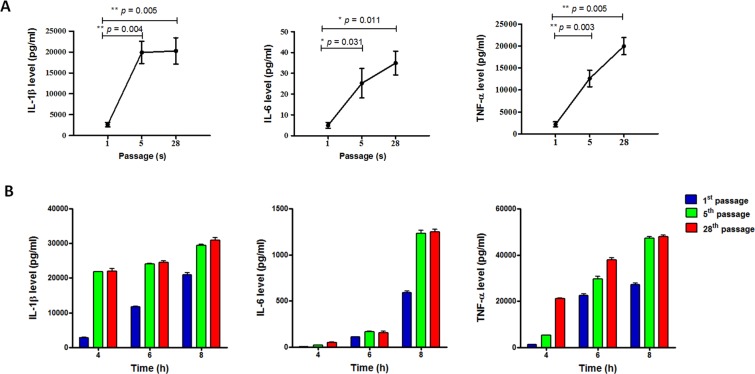
Activation of cytokine production by THP-1 cells in response to infection with passaged *B. pseudomallei* strain HBPUB15305A. (**A**) The levels of IL-1β, IL-6, and TNF-α production from THP-1 cells infected with the first, fifth, and 28^th^ passaged *B. pseudomallei* strain HBPUB15305A. (**B**) The levels of IL-1β, IL-6, and TNF-α by THP-1 cells in response to infection with the first, fifth, and 28^th^ passaged *B. pseudomallei* strain HBPUB15305A at different time points. Asterisks indicate significant differences (*p < 0.05 and **p < 0.01) between groups. Error bars represent standard errors of the means for experiments performed in triplicate.

In proteomic analyses of *B. pseudomallei* strain HBPUB15305A, the upregulated proteins included porin, flagellar biosynthesis protein, and serine protease. These proteins have been reported to play roles in immune activation, causing increases in pro-inflammatory cytokine production. Thus, we assessed the abilities of *B. pseudomallei* strain HBPUB15305A to elicit cytokine production from THP-1 cells, following infection with bacteria prepared from the first, fifth, and 28^th^ passages. THP-1 cells were infected with passaged *B. pseudomallei* at a multiplicity of infection (MOI) of 10. Culture supernatants were harvested at various time points over the course of 8 h to measure the release of interleukin (IL)-1β, IL-6, and tumor necrosis factor (TNF)-α. The cytokine production of THP-1 cells at 4 h after infection with the passaged *B. pseudomallei* strain HBPUB15305A is presented in Fig. 8A; IL-1β, IL-6, and TNF-α cytokine levels increased following infection with bacteria prepared from both the fifth and 28^th^ passages. The levels of IL-1β, IL-6, and TNF-α were initially detected at 4 h after infection and gradually increased in a time-dependent manner (Fig. 8B). This implied that *B. pseudomallei* is able to modify its antigens during serial passage, thus influencing host immune activation.

## Discussion

Our study demonstrated phenotypic changes during *in vitro* serial passage of *B. pseudomallei* fresh clinical isolates and reference strain K96243. Notably, the pathogenesis of *B. pseudomallei* was enhanced after *in vitro* serial passage in bacteria prepared from the fifth passage in both *B. pseudomallei* strain HBPUB15305A and reference strain K96243. These results provide evidence that supports a link between serial passage and *B. pseudomallei* virulence. This is consistent with the results from Mankoski *et al*., who found an increase in *H. pylori* virulence after passaging in piglets^10^. The passaged *H. pylori* exhibited increased expression of the flagellin gene, as well as increased bacterial colonization^10^. However, Kim *et al*. reported reductions in adhesion rates, motility, and cytotoxicity following repeated passages of *H. pylori*; they also found that *in vitro* passage reduced *H. pylori* virulence in gerbils^12^. Moreover, Somerville *et al*. demonstrated a reduction in aconitase activity of *Staphylococcus aureus* after serial passage for 6 weeks^11^. Aconitase is an enzyme that regulates virulence factor production in *S. aureus*; thus, reduced aconitase activity may influence the virulence of passaged *S. aureus*^11^. In addition, the effects of *in vitro* serial passage on genomic and phenotypic changes in *Mycobacterium tuberculosis* have been reported^13^. After *M. tuberculosis* was passaged for 4 weeks, the bacteria exhibited a reduced ability to synthesize the cell wall lipid PDIM, which is an important virulence factor. Whole genome microarray revealed that PDIM loss resulted from the deletions of *pps* and *drr* operons^13^. Thus far, many studies have shown that the virulence levels of some bacteria are reduced during serial passage in the laboratory. However, our findings demonstrated increased virulence in *B. pseudomallei* following serial passage. We presume that *B. pseudomallei* possess various adaptive mechanisms for the modulation of protein expression, allowing alterations of its virulence during serial passage.

To better understand the mechanisms underlying changes in adaptation and virulence of passaged *B. pseudomallei*, proteomic studies were performed. Proteomic profiles of *B. pseudomallei* prepared from the fifth and 28^th^ passages of *B. pseudomallei* strain HBPUB15305A and reference strain K96243 were determined by two-dimensional electrophoresis and LC-MS/MS, in comparison with the proteomes of the respective bacteria prepared from the first passage. The resulting proteome data provided information regarding differential protein expression patterns of *B. pseudomallei* following serial passage. Notably, the numbers of differentially expressed proteins increased with increasing number of passages. In addition, proteomic results were validated using qRT-PCR. The mRNA expression analyses confirmed our proteomic results. In bacteria prepared from the 28^th^ passage, 11 upregulated proteins were observed in both *B. pseudomallei* strain HBPUB15305A and reference strain K96243. These proteins were involved in *B. pseudomallei* metabolic processes, energy production, cell division, and virulence; these proteins may help to maintain bacterial survival and growth during long-term passage. In contrast, 31 upregulated proteins were observed only in *B. pseudomallei* strain HBPUB15305A, while 35 upregulated proteins were observed only in reference strain K96243. These results indicated that protein expression profiles differed between the *B. pseudomallei* strain HBPUB15305A and reference strain K96243 after serial passage.

Our proteomic profiles provided the information regarding potential underlying mechanisms of *B. pseudomallei* adaptation during *in vitro* serial passage. Several differentially expressed proteins that play important roles in *B. pseudomallei* virulence were observed. In passaged *B. pseudomallei* strain HBPUB15305A, the flagellar biosynthesis protein was upregulated; this protein is involved in flagellin synthesis. Flagellin has been reported to facilitate *B. pseudomallei* invasion into host cells^14^. We presume that increased flagella expression might enhance *B. pseudomallei* invasion ability. Similarly, Mankoski *et al*. studied the effect of *in vivo* passage in piglets, with respect to the colonization of *H. pylori*; they found that bacterial colonization was increased after an increased number of passages, which was consistent with increased expression of the *filA* gene required for flagellin synthesis^10^.

Additionally, our study reported upregulation of flavohemoprotein in the passaged *B. pseudomallei* strain HBPUB15305A. Flavohemoprotein is reportedly involved in the detoxification of nitric oxide in *Escherichia coli*^15^. In the *G. mellonella* killing assay, the mortality rate was increased in *G. mellonella* larvae that were infected with passaged *B. pseudomallei*. It is likely that flavohemoprotein detoxifies nitric oxide produced by *G. mellonella* larvae as a defense mechanism against microbial infection. Moreover, we found that porin, an outer membrane protein, was upregulated in bacteria prepared from the 28^th^ passage of *B. pseudomallei* strain HBPUB15305A and the fifth passage of reference strain K96243. Previously, porin was reported to play an important role in *B. pseudomallei*, acting as efflux pump and causing antibiotic resistance^16^. In addition, it has been reported to play crucial roles in membrane stability and interaction with the environment^17^. These findings suggest that porin helps passaged *B. pseudomallei* to stabilize outer membrane structure to tolerate environmental change during serial passage.

Upregulation of serine protease was detected in the passaged *B. pseudomallei* strain HBPUB15305A. Generally, serine protease interacts with denatured and misfolded periplasmic proteins, limiting cell damage under stress conditions (stress tolerance), and acts as an extracellular protease^18,19^. In its role as an extracellular protease, serine protease has been shown to cause extensive damage to mammalian physiological proteins and is involved in the pathogenesis of melioidosis^19,20^. Thus, serine protease may be an important bacterial component that contributes to *B. pseudomallei* virulence during serial passage. In addition, ferritin was upregulated in the passaged *B. pseudomallei* strain HBPUB15305A. Ferritin has been reported to serve as an alternate iron scavenger for *B. pseudomallei* growth and intracellular survival^21^. Perhaps during serial passage, ferritin delivers iron to facilitate intracellular survival, thus contributing to the virulence of *B. pseudomallei*. Furthermore, serine protein kinase was upregulated in bacteria prepared from the 28^th^ passages of *B. pseudomallei* strain HBPUB15305A and reference strain K96243; this protein has a role in sensing the environment and subverting host defense processes, and is a virulence factor in many pathogens (e.g., *Yersinia* spp., *Staphylococcus* spp., and *Salmonella* spp.)^22^. In *B. pseudomallei*, other kinases (e.g., polyphosphate kinase) are involved in biofilm formation, motility, and stress response^23^. There is minimal information regarding the role of serine protein kinase in *B. pseudomallei* virulence. However, serine protein kinase was presumably upregulated in response to environmental change during long-term passage, which might have been advantageous for *B. pseudomallei* virulence. Oxidoreductase, an antioxidant enzyme^24^, was also upregulated in the passaged reference strain K96243. This enzyme might aid in pathogen survival under stress conditions, such as oxidative stress, and may thus facilitate *B. pseudomallei* survival during serial passage.

Notably, Tex protein was upregulated in both the passaged *B. pseudomallei* strain HBPUB15305A and reference strain K96243. Tex is a bacterial toxin regulator in several bacteria, including *Bordetella pertussis*^25^*, Clostridium perfrigens*^26^ and *B. pseudomallei*^27^. Moule *et al*. reported that Tex was involved in *B. pseudomallei* virulence and may be a useful vaccine candidate^27^. Our findings suggested that an increase in Tex expression may contribute to *B. pseudomallei* virulence during serial passage. Taken together, the proteomic changes provide information regarding adaptation mechanisms of passaged *B. pseudomallei*, which contribute to increased virulence.

Serial passage is known to alter host immune activation. In *M. tuberculosis*, there was a reduction in immune activation during serial passage, due to the deletion of key protective antigens^28^. However, there has been no report regarding the effect of serial passage of *B. pseudomallei* on the host immune response. Pro-inflammatory cytokines plays a critical role in regulation of host immune responses^29,30^; these cytokines were associated with disease outcome during *B. pseudomallei* infection and have been extensively investigated to understand the immune response to and pathogenesis of the pathogen. Thus, the levels of pro-inflammatory cytokines (IL-1β, IL-6, and TNF-α) were examined in this study. Our results indicated increased cytokine production in response to infection with the passaged *B. pseudomallei* strain HBPUB15305A, compared with bacteria prepared from the first passage. We hypothesized that upregulated proteins during *in vitro* serial passage may play roles in immune activation, which might be related to increased production of pro-inflammatory cytokines. Our proteomic analysis showed that, of the upregulated proteins, there may have an effect on the immune response to the passaged *B. pseudomallei* strain HBPUB15305A. Flagellin, an immunodominant antigen^14^, has been found to induce the production of inflammatory cytokines in response to *B. pseudomallei* infection^31^; porin has been reported to play a role in immune activation in response to *B. pseudomallei* infection^17^. In addition, serine protease has been demonstrated to activate the host immune response due to its antibody-binding epitope^32^. For *B. pseudomallei* reference strain K96243, our proteomic analysis demonstrated upregulation of proteins such as chaperone GroEL and elongation factor TU; these proteins may be involved in cytokine production because they are reportedly immunogenic *B. pseudomallei* proteins^33,34^.

During long-term serial passage, bacteria encounter a variety of stresses, including nutrient limitation, oxidative stress, and change in pH level. In our study, stress response proteins, including chaperone GroL, chaperone GroEL, and elongation factor TU were upregulated in the passaged *B. pseudomallei* reference strain K96243. These proteins play roles in stress responses and help *B. pseudomallei* to survive in stress conditions^35^. Furthermore, these stress response proteins have been reported to play roles in the virulence of *B. pseudomallei*^35^. Upregulation of these proteins may contribute to pathogenesis during the infection of HeLa cells and *G. mallonella* larvae by the passaged *B. pseudomallei* reference strain K96243.

Importantly, alterations in metabolic proteins were observed in the passaged *B. pseudomallei* strain HBPUB15305A and reference strain K96243. Our findings suggested that these upregulated metabolic proteins maintained bacterial survival during serial passage. KEGG pathway analysis showed upregulation of proteins in central metabolic pathways, including pentose phosphate, lipid metabolism, fatty acid degradation, amino acid biosynthesis, and amino acid metabolism. In a similar LC-MS/MS analysis, Leiser *et al*. reported that proteins involved in amino acid transport and metabolism (glutamate dehydrogenase and glutamine synthetase) were upregulated in *Yersinia pestis* after 60 serial passages^36^.

The ability of *B. pseudomallei* to adapt its metabolic pathways for survival was demonstrated in a previous report^37^. Challacombe *et al*. demonstrated that *B. pseudomallei* could utilize a wide variety of substrates, including carbohydrates, lipids, glycolipids, dicarboxylic acids, and amino acids during host cell infection. This indicated the capacity of *B. pseudomallei* to adjust its metabolism for survival in the presence of a variety of carbon sources^37^. In our study, we also observed altered expression of metabolic proteins that play major roles in carbohydrate and amino acid metabolic pathways in both passaged *B. pseudomallei* strains. These changes may have helped facilitate survival of *B. pseudomallei* during serial passage.

Previous studies have described associations between adaptive metabolism and pathogenesis in intracellular pathogens such as *Listeria monocytogenes*, *Shigella flexneri*, *Salmonella* spp., *M. tuberculosis*, and *B. pseudomallei*^37–42^; these findings are consistent with our proteomic results. Arginine deiminase was upregulated in both the passaged *B. pseudomallei* strain HBPUB15305A and reference strain K96243; this is an enzyme involved in amino-acid metabolism, which may allow passaged *B. pseudomallei* to use amino acids as an energy source, thus enhancing bacterial survival. Moreover, arginine deiminase may contribute to bacterial survival by increasing acid tolerance during continuous culture in LB medium^43^.

In conclusion, we demonstrated the adaptive survival of *B. pseudomallei* during serial passage in LB medium. Our results showed phenotypic changes in *B. pseudomallei*, including in the virulence and immune activation characteristics of a fresh clinical isolate and reference strain K96243 during both short-term and long-term passage. Moreover, our proteomic and transcriptomic data provide supporting information regarding the adaptive mechanisms of the pathogen during *in vitro* serial passage. These data will allow researchers to consider phenotypic adaptations that occur during serial passage of *B. pseudomallei*, which may affect the conclusions based on data obtained from passaged bacteria. Although numbers of plaque-forming unit caused by bacteria before the fifth passage seemed to be increased, there were no significant differences when comparing to the first passage. This indicated that the serial passages have strong impact on plaque formation since the fifth passage. Therefore, we suggest that *B. pseudomallei* should be limited to five serial passages to avoid phenotypic adaptations.

## Materials and methods

### Ethics statement

All experiments and methods were performed in accordance with relevant guidelines and regulations. This project has been approved from the ethics committee of Faculty of Tropical Medicine, Mahidol University, Bangkok, Thailand (Reference No: MUTM 2018-043-01). All blood cultures positive for *B. pseudomallei* were anonymised. The committees waived the requirement to obtain individual informed consent due to the anonymity and minimal risk to the subjects.

The *B. pseudomallei* wax moth larvae infection protocol was approved by the Animal Ethics Committee, Mahidol University, Bangkok, Thailand (Reference: FTM-ACUC 020/2561) based on the ethics of animal experimentation guidelines of the National Research Council of Thailand.

### Biosecurity aspects

Both animal and general bacterial laboratory facilities were operated following all the security and safety regulations of our university. Animal experiments were carried out at Faculty of Tropical Medicine, Mahidol University under the national procedure for infectious agents. This is a BSL2 plus facility that is currently being upgraded to BSL3 practices.

This project has been approved from the biosafety committee of Faculty of Tropical Medicine, Mahidol University, Bangkok, Thailand (Reference No: FTM-IBC-18-01 and FTM-IBC-18-02).

### Bacterial strains and growth condition

Five blood cultures positive for *B. pseudomallei* (HBPUB15305A, HBPUB15309A, HBPUB15310B, HBPUB15318A, and HBPUB15322B) from five patients who were newly diagnosed with melioidosis were obtained from Sappasithiprasong Hospital, Ubon Ratchathani, Thailand. *B. pseudomallei* reference strain K96243 was obtained from a laboratory stock in the Department of Microbiology and Immunology, Faculty of Tropical Medicine, Mahidol University, Bangkok, Thailand. For *in vitro* serial passages, *B. pseudomallei* fresh clinical isolates and reference strain K96243 were grown overnight on LB (Becton Dickinson, Sparks, MD, USA) plates. In this study, five fresh clinical isolates of *B. pseudomallei* were directly cultured from blood samples with zero prior passages. Then, a single colony from each strain was inoculated into 10 ml fresh LB broth and incubated at 37 °C in a shaking incubator at 200 rpm for 18 h. Serial passage was daily performed by transferring 100 µl of overnight bacterial culture into 10 ml fresh LB broth, and maintained in a 37 °C shaking incubator at 200 rpm. The bacteria were freshly passaged in LB broth for 28 days. At 18 h culture, passaged *B. pseudomallei* fresh clinical isolates and reference strain K96243 were collected, washed with phosphate-buffered saline (PBS), and subjected to bacterial pathogenesis, immune activation, proteome, and transcriptome studies.

### Bacterial growth analysis

To determine growth of *B. pseudomallei* fresh clinical isolates, optical density of cultures at various time points was recorded. In brief, overnight culture *B. pseudomallei* isolates were washed with sterile PBS and adjusted in LB broth to obtain bacterial suspensions of approximately 1×10^6^ CFU/ml. 0.1 ml of these suspension were added to 10 ml of LB broth and incubated at 37 °C in air with shaking at 200 rpm for 24 h. At 2, 4, 6, 12, and 18 h, serial dilution was performed to determine the number of colony forming unit counts (CFUs).

### Plaque formation assay

Plaque-forming efficiency was assessed as previously described^44^. Human cervix carcinoma (HeLa) cell line was obtained from the American Type Culture Collection (ATCC, Manasssas, VA). HeLa cells were routinely maintained in Dulbecco’s modified Eagle medium (DMEM) (Gibco, Grand Island, N.Y., U.S.A.) with 10% (v/v) heat-inactivated fetal bovine serum (FBS) and cultured in 5% CO_2_ atmosphere at 37 °C in humidified incubator. HeLa cells were infected with passaged *B. pseudomallei* at MOI of 20, and incubated at 37 °C with 5% CO_2_ for 2 h. Thereafter, the infected cell monolayers were washed, and replaced with medium-containing kanamycin (250 μg/ml). The plates were incubated at 37 °C in a humidified 5% CO_2_ atmosphere for 21 h. Plaques were stained with 1% (w/v) crystal violet to enhance plaque visualization. The numbers of plaques were counted under microscope. Plaque-forming efficiency was calculated by the following equation: number of plaques/ CFUs of bacteria added per well.

### Invasion and intracellular survival assay

Invasion and intracellular survival assays were modified from protocols described by Techawiwattanaboon *et al*.^45^. HeLa cells were seeded approximately 1.5×10^6^/well in 6-well plates. Passaged *B. pseudomallei* (the first, fifth, and 28^th^ passages) were used to infect HeLa cells at MOI of 20 and incubated at 37 °C with 5% CO_2_ for 2 h. After 2 h incubation, the infected cell monolayers were washed with PBS and replaced with medium containing kanamycin (Sigma-aldrich, MO, USA) (250 μg/ml) to eliminate extracellular bacteria. The infected HeLa cells were washed with PBS and subsequently lysed with 0.1% Triton X-100 (Sigma-aldrich, MO, USA) at 2, 4, 6, 12, and 18 h post-infection. Viable invading and intracellular bacteria were quantitated by plating serial ten-fold dilutions of lysates on LB agar and counting CFUs after 36 h of incubation at 37 °C.

### Determination of cytokine production

Human acute monocytic leukemia cell line THP-1 (ATCC TIB-202) was routinely maintained in Roswell Park Memorial Institute (RPMI) 1640 medium (Gibco, Grand Island, N.Y., USA) supplemented with 10% FBS (Thermo Scientific, MA, US) at 37 °C in 5% CO_2_ in humidity incubator. THP-1 cells were plated at 1×10^5^/ml in 24-well plate, and induced with 50 ng/ml Phorbol 12-myristate 13-acetate (PMA) for 36 h to differentiate into macrophage-like cells. PMA-stimulated THP-1 cells were infected with passaged *B. pseudomallei* at MOI of 10. The supernatant was collected at various time points (2, 4, and 8 h), and analyzed for IL-1β, IL-6, and TNF-α release by enzyme-linked immunosorbent assay (ELISA) (BD OptEIA, San Diego, USA) according to manufacturer’s instructions. The absorbance at 450 nm was monitored by SunriseTM Absorbance Reader (Tecan, Switzerland). The average OD values of triplicate wells were used for analysis. Three independent experiments were performed.

### *Galleria mellonella* killing assay

*G. mellonella* killing assays were performed as previously described^46^, with some modifications. Ten larvae were used; all were 2–2.5 cm in length, 250–300 mg in body weight, and free of melanization. After 18 h of growth, passaged *B. pseudomallei* were adjusted to a concentration of 100 CFUs in PBS. A Hamilton syringe was used to inject a 10 µl aliquot of the bacterial suspension into the body cavity of a *G. mellonella* larva via the proleg. Each control larva was injected with 10 µl of PBS. Following injection, larvae were incubated in the dark at 37 °C. At 24, 30, 36, and 40 h post-injection, larvae were individually examined for pigmentation and mobility. Larvae were considered dead when they displayed no movement in response to gentle prodding with a pipette tip. The numbers of dead larvae and times of death were recorded.

### Proteomic analysis

Protein extraction of the first, fifth, and 28^th^ passaged *B. pseudomallei* strain HBPUB15305A and reference strain K96243 were performed. The selected bacterial passages were centrifuged at 8,000 xg for 1 min at 4°C. The bacterial pellets were washed once with cold PBS and then resuspended in 1 ml of cold 2D lysis buffer containing 8 M urea, 2 M thiourea, 4% 3-((3-cholamidopropyl) dimethylammonio)-1-propanesulfonate (CHAPS), and 50 mM Dithiothreitol (DTT). The suspension was sonicated on ice at 20% amplitude for 3 min. The cell lysate was centrifuged at 12,000 xg for 3 min at 4 °C. The supernatant was collected and stored at −80 °C until use.

Two-dimensional gel electrophoresis (2-DE) was performed as previously described^47^, with some modifications. Protein samples from the first, fifth, and 28^th^ passages of *B. pseudomallei* strain HBPUB15305A and reference strain K96243 were cleaned using the 2D clean-up kit (GE Healthcare, Germany), and the concentration was determined using Bradford kit (Bio-Rad, USA) and bovine serum albumin was used as a protein standard. Fifty micrograms of protein samples from each passage were separated by isoelectric focusing on immobiline dry strip, 7-cm IPG strip pH 3-10 NL and then subjected to second dimensional separation using 12% acrylamide gel. Separated proteins were stained with SYPRO Ruby fluorescent stain (Bio-Rad, USA). Gel images were captured using Typhoon scanner model typhoon trio serial number 1557771 (GE Healthcare, USA). ImageMaster 2D Platinum version 5.0 (GE Healthcare, USA) was used for matching and analysis of protein spots on 2D gels. The fold changes in the intensity of matched protein spots between 2 groups; the first and fifth passages and the first and 28^th^ passages were determined. Protein spots with differential expression of more than 1.5-fold change and *p* < 0.05 when tested with ANOVA were selected, visualized with silver stain and then subjected to protein identification using liquid chromatography and tandem mass spectrometry (LC-MS/MS).

In-gel tryptic digestion and protein identification were performed as previously described^47^. Selected proteins were excised from gel followed by tryptic digestion. The gel pieces were destained using 50% acetonitrile in 50 mM ammonium bicarbonate and gel slices from 2DE were destained using 30 mM potassium ferricyanide and 100 mM sodium thiosulfate until colorless. Gel pieces were incubated in 4  mM DTT at 60 °C for 15 min. Proteins were alkylated by adding 250 mM iodoacetamide and incubating at room temperature in the dark for 30 min. The reaction was quenched with 4 mM DTT and dehydrated in 100% acetonitrile. Gel pieces were then rehydrated with 10 ng/μL trypsin in 50 mM ammonium bicarbonate at 37 °C overnight. Acetonitrile was added to extract peptides. The supernatant was collected and the peptide mixtures were completely dried by speed-vac (Eppendorf, Hamburg, Germany).

Tryptic-digested samples were resuspended in 0.1% formic acid containing 2% acetonitrile and then introduced to an UltiMate 3000 nano-LC system (Dionex, Surrey, UK) coupled with a micrOTOF-Q (Bruker Daltonics, Bremen, Germany) to analyze digested proteins. Data acquisitions were controlled using Hystar soft-ware (Bruker Daltonics, Bremen, Germany). MS and MS/MS spectra covered the mass range of *m/z* 400–2000 and *m/z* 50–1500, respectively. LC–MS/MS data files were searched using Mascot version 2.4.1 (Matrix Science, London, UK) against the NCBInr database. Protein identification was accepted at 95% confidence. All identified proteins were classified based on their functions using UniProt database. The differentially expressed proteins were subjected to pathway analysis using KEGG database^48^, and analyzed the heat maps with heatmapper^49^.

### Transcriptomic analysis

RNA was isolated from stationary phase growth of passaged *B. pseudomallei* cells grown at 37 °C for 18 h by adding RNAprotect bacterial reagent (Qiagen, Germany) to bacterial culture and incubating for 5 min at room temperature. Subsequently, total RNA was extracted from the bacterial pellets using Trizol (Invitrogen, Carlsbad, CA, USA) and RNeasy kit (Qiagen, Germany). Contaminant genomic DNA was removed using DNase I (New England Biolabs, United States). The following genes; *arcA, ef-tu, flhF, groEL, groL, hmp, omp, stk, tex, tftC, BPSL2863*, and *BPSS0962* were selected for validation of proteomic data. Primer sequences are shown in Supplementary Tables S5. Real-time RT-PCR was performed using KAPA SYBR fast one-step (Kapabiosystems, Wilmington, Massachusetts, USA) with following conditions: reverse transcription at 42 °C for 5 min, enzyme activation at 95 °C for 3 min, then 40 cycles of denaturation at 95 °C for 3 s, annealing at 55 °C for 30 s, and melting curve analysis at 65 to 95 °C, increment 0.5 °C for 5 s in a CFX96 Touch Real-Time PCR Detection System (Bio-Rad, Singapore). Relative mRNA levels were determined by fold change in expression, calculated by 2^−ΔΔCT^ using the relative mRNA level of 23S RNA, as a baseline for comparison.

### Statistical analysis

All assays were conducted in triplicate. Analysis of variance, general linear modeling, and Tukey’s *post-hoc* by NCSS 11 statistical software 2016 (NCSS, Kaysville, Utah, USA), were used to test statistical differences for plaque-forming efficiency. Paired *t*-tests were performed using GraphPad Prism version 6.02 (GraphPad, La Jolla, CA, USA) to compare differences in invasion and intracellular assays, as well as in cytokine production. For the *G. mellonella* killing assay, a log-rank (Mantel–Cox) test by GraphPad Prism was used to compare survival curves.

## Supplementary information

Supplementary Information.
