# Supplementary material for: In vitro passage alters virulence, immune activation and proteomic profiles of Burkholderia pseudomallei
Source: Sci Rep. 2020 May 20;10:8320. doi: 10.1038/s41598-020-64914-4 (PMC7239947; doi:10.1038/s41598-020-64914-4)
Supplement: Supplementary file 1 — Supplementary Information. [file 41598_2020_64914_MOESM1_ESM.pdf]

In vitro passage alters virulence, immune activation and proteomic profiles of *Burkholderia pseudomallei*

Taksaon Duangurai[1, 2], Onrapak Reamtong[3], Amporn Rungruengkitkun[1], Varintip Srinon[4], Usa Boonyuen[3], Direk Limmathurotsakul[5, 6], Narisara Chantratita[1, 5], Pornpan Pumirat[1*]

[1]Department of Microbiology and Immunology, Faculty of Tropical Medicine, Mahidol University, Bangkok, Thailand.

[2]Department of Companion Animal Clinical Sciences, Faculty of Veterinary Medicine, Kasetsart University, Bangkok, Thailand.

[3]Department of Molecular Tropical Medicine and Genetics, Faculty of Tropical Medicine, Mahidol University, Bangkok, Thailand.

[4]Faculty of Veterinary Science, Veterinary Diagnostic Center, Mahidol University, Nakhon Pathom, Thailand.

[5]Mahidol Oxford Tropical Medicine Research Unit, Faculty of Tropical Medicine, Mahidol University, Bangkok, Thailand.

[6]Department of Tropical Hygiene, Faculty of Tropical Medicine, Mahidol University, Bangkok, Thailand.

*Corresponding author

E-mail: pornpan.pum@mahidol.ac.th, angoonppu@gmail.com

Supplementary information

- Supplementary Figure S1
- Supplementary Figure S2
- Supplementary Table S1
- Supplementary Table S2
- Supplementary Table S3
- Supplementary Table S4
- Supplementary Table S5

Supplementary Figure S1. Heat map of differentially expressed proteins (ANOVA, $p < 0.05$) that showed at least 1.5 fold change in the first, fifth, and 28th passaged *B. pseudomallei* strain HBPUB15305A in LB broth, incubated 18 h at 37°C in shaking incubator. Red indicates increase of protein levels while green represents repression according to the color scale at the top of the figure.

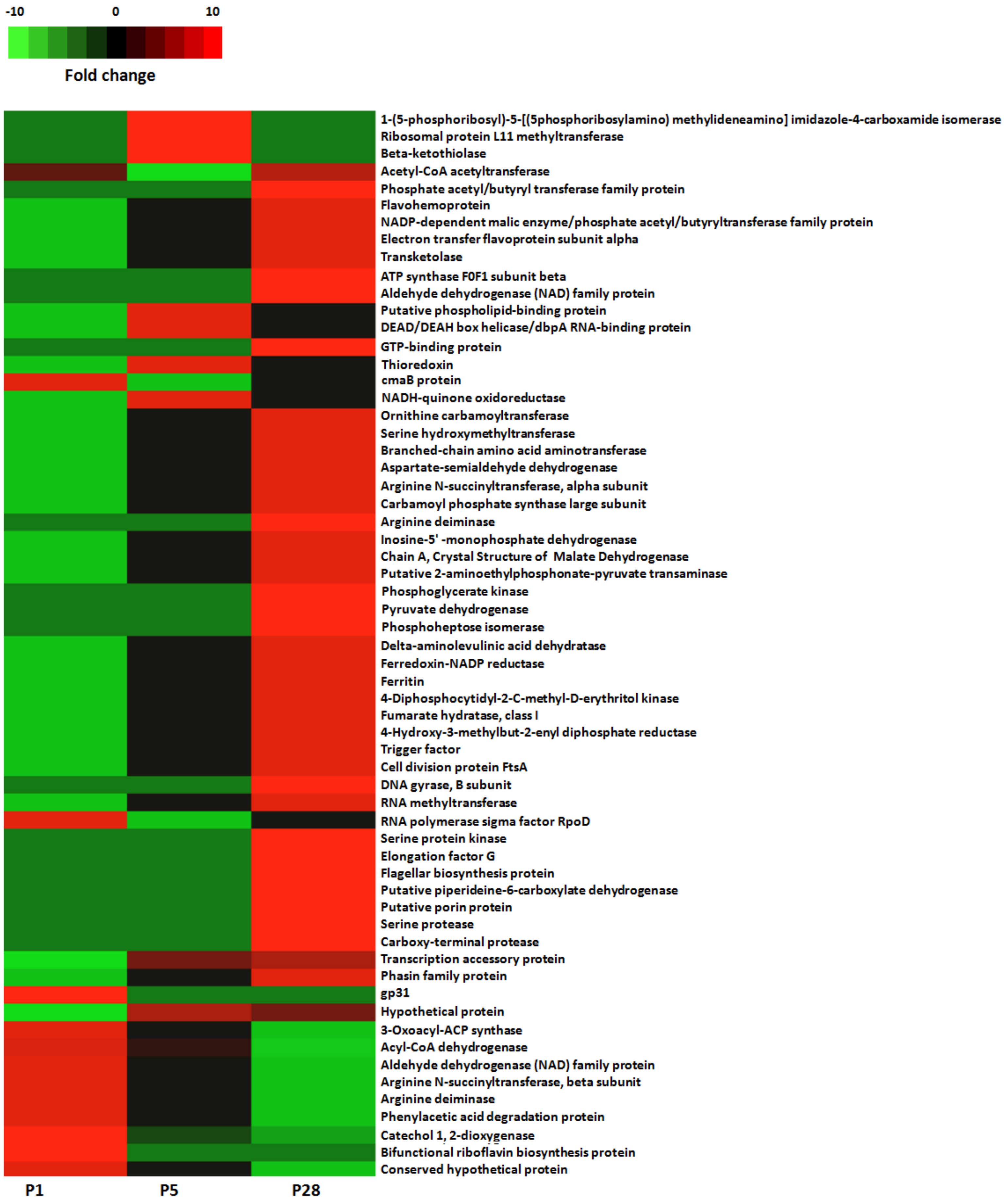

Supplementary Figure S2. Heat map of differentially expressed proteins (ANOVA, $p < 0.05$) that showed at least 1.5 fold change in the first, fifth, and 28th passaged *B. pseudomallei* reference strain K96243 in LB broth, incubated 18 h at 37°C in shaking incubator. Red indicates increase of protein levels while green represents repression according to the color scale at the top of the figure.

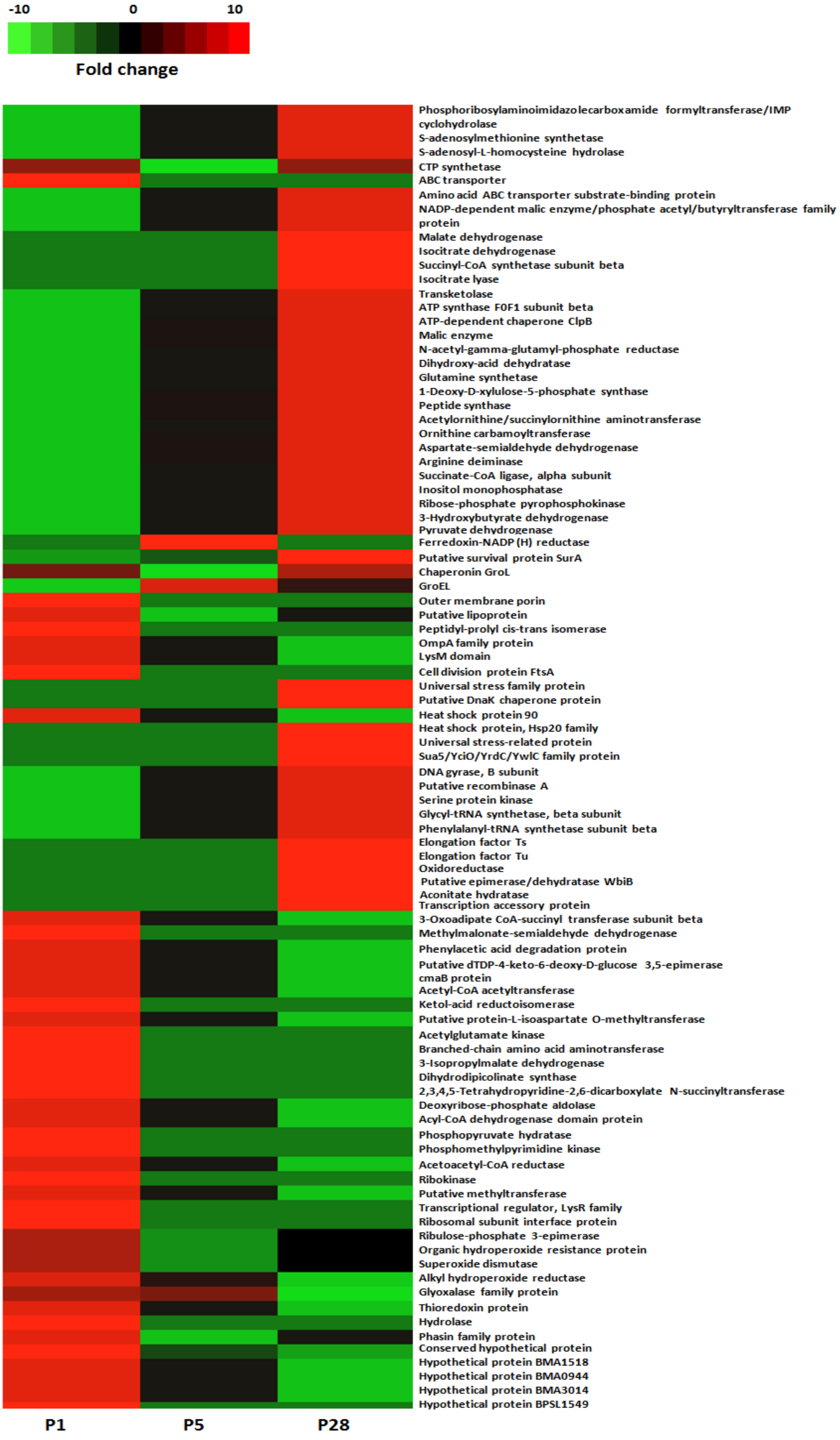

Table S1. Summary of altered-proteins of the fifth passage of *B. pseudomallei* strain HBPUB15305A compared to the first passage in LB broth, incubated 18 h at 37°C in shaking incubator.

Spot no.	Altered proteins	Accession number	Score	Mass (Da)	pI	Fold change	Biological functions
**Up-regulated proteins of the fifth passage compared to the first passage**							
**Metabolic enzymes**							
1	1-(5-phosphoribosyl)-5-[(5phosphoribosylamino) methylideneamino] imidazole-4-carboxamide isomerase	gi|53724261	848	26572	4.37	1.89	Histidine biosynthesis
3	Acyl-CoA dehydrogenase	gi|126227018	620	63614	5.52	1.70	Acyl-CoA dehydrogenase activity,<br>Flavin adenine dinucleotide binding
4	Ribosomal protein L11 methyltransferase	gi|237503463	73	32878	4.65	1.56	Methyltransferase activity
6	Beta-ketothiolase	gi|53723565	197	48319	6.56	1.73	Transferase activity
**Binding proteins**							
5	Putative phospholipid-binding protein	gi|126230840	240	25789	6.36	1.55	Binding protein
8	DEAD/DEAH box helicase/dbpA RNA-binding protein	gi|126231871	50	50927	5	6.39	RNA binding protein
**Antioxidants**							
7	Thioredoxin	gi|225935117	490	31093	4.59	1.80	Cell redox homeostasis
9	NADH-quinone oxidoreductase	gi|779726438	84	29325	5.07	1.60	ATP synthesis coupled
**Virulence factor**							
2	Transcription accessory protein	gi|53723516	105	84505	6.13	1.53	Nucleic acid binding,<br>Nucleobase-containing compound metabolic process
**Hypothetical protein**							
10	Hypothetical protein	gi|126228446	276	19871	4.48	1.76	-
**Down-regulated proteins the fifth passage compared to the first passage**							
**Metabolic enzymes**							
**A. Lipid metabolism**							
11	Catechol 1,2-dioxygenase	gi|52213327	1250	32947	5.62	-1.64	Catechol-containing compound catabolic process

Spot no.	Altered proteins	Accession number	Score	Mass (Da)	pI	Fold change	Biological functions
16	Bifunctional riboflavin biosynthesis protein	gi|126227043	116	40576	5.58	Absent	Riboflavin biosynthesis
17	Acetyl-CoA acetyltransferase	gi|53724635	221	40889	6.67	Absent	Acetyl transferase
**Transcription initiation factor**							
13	RNA polymerase sigma factor RpoD	gi|52213190	32	76698	4.87	-1.70	Transcription initiation
**Antioxidant**							
12	cmaB protein	gi|126229582	449	35383	5.77	-1.95	Polyketide biosynthesis
**Hypothetical protein**							
15	Hypothetical protein	gi|126226827	426	27042	9.3	Absent	-
**Miscellaneous**							
14	gp31	gi|17978628	199	39340	5.75	Absent	Protein folding

Table S2. Summary of altered-proteins of the 28th passage of *B. pseudomallei* strain HBPUB15305A compared to the first passage in LB broth, incubated 18 h at 37°C in shaking incubator.

Spot no.	Altered proteins	Accession number	Score	Mass (Da)	pI	Fold change	Biological functions
**Up-regulated proteins of the 28th passage compared to the first passage**							
**Metabolism**							
**A. Lipid metabolism**							
1<br>2	Acetyl-CoA acetyltransferase	gi|184210116	2093	40488	6.62	1.95	Acetyltransferase
3	Phosphate acetyl/butyryl transferase family protein	gi|126232338	1963	48998	5.82	2.27	Fatty acid biosynthetic process, oxidation-reduction process
**B. Energy production and conversion**							
4	Flavohemoprotein	gi|157808924	1988	43498	6.1	8.91	Response to nitrosative stress, Binding protein
5<br>6	NADP-dependent malic enzyme/phosphate acetyl/butyryltransferase family protein	gi|126226702	1174	83153	5.81	1.97	Malate metabolic process
7	Electron transfer flavoprotein subunit alpha	gi|53724769	2278	31623	4.86	1.92	Fatty acid beta-oxidation using acyl-CoA dehydrogenase
8	Transketolase	gi|714840505	871	71541	6.03	2.37	Iron binding
9	ATP synthase F0F1 subunit beta	gi|53724016	2693	50591	5.26	only P28	ATP synthesis coupled proton transport
10	Aldehyde dehydrogenase (NAD) family protein	gi|126229915	506	54739	6.11	only P28	Oxidoreductase activity
11	GTP-binding protein	gi|53724833	807	67456	5.35	only P28	Binding protein
**C. Amino acid transport and metabolism**							
13<br>14	Ornithine carbamoyltransferase	gi|157936484	2175	37993	6.17	3.51	Arginine biosynthetic process<br>Arginine catabolic process to ornithine

Spot no.	Altered proteins	Accession number	Score	Mass (Da)	pI	Fold change	Biological functions
15	Serine hydroxymethyltransferase	gi|53725619	764	44957	6.37	10.16	Glycine biosynthetic process from serine<br>Tetrahydrofolate interconversion
16	Branched-chain amino acid aminotransferase	gi|53724736	944	33964	6.1	1.88	Isoleucine biosynthetic process, leucine biosynthetic process, valine biosynthetic process
17	Aspartate-semialdehyde dehydrogenase	gi|126230344	880	39869	5.32	2.20	'de novo' L-methionine biosynthetic process , diaminopimelate biosynthetic process, isoleucine biosynthetic process, lysine biosynthetic process via diaminopimelate, threonine biosynthetic process
18	Arginine N-succinyltransferase, alpha subunit	gi|126226303	889	38110	5.3	2.66	Arginine catabolic process
19	Carbamoyl phosphate synthase large subunit	gi|53725563	2037	118167	5.12	1.77	'de novo' UMP biosynthetic process<br>Arginine biosynthetic process
20	Arginine deiminase	gi|53723412	2701	46022	5.57	only P28	Arginine catabolic process to ornithine
21	Inosine-5' -monophosphate dehydrogenase	gi|53723710	2471	51866	7.21	2.01	GMP biosynthetic process
22	Chain A, Crystal Structure of Malate Dehydrogenase	gi|189339693	2257	35291	5.71	3.82	Metabolic process<br>Tricarboxylic acid cycle
23	Putative 2-aminoethylphosphonate-pyruvate transaminase	gi|126230452	1234	38257	6.08	2.49	Organic phosphonate catabolic process
24	Phosphoglycerate kinase	gi|53726131	1309	41177	5.4	only P28	Glycolytic process
25	Pyruvate dehydrogenase	gi|53723814	42	100265	5.63	only P28	Acetyl-CoA biosynthetic process

Spot no.	Altered proteins	Accession number	Score	Mass (Da)	pI	Fold change	Biological functions
26	Phosphoheptose isomerase	gi|53725822	679	20771	5.8	only P28	Capsule polysaccharide biosynthetic process D-glycero-D-manno-heptose 7-phosphate biosynthetic process
**D. Inorganic ion transport and metabolism**							
27	Delta-aminolevulinic acid dehydratase	gi|53723824	1040	39353	5.51	2.76	Porphyrin biosynthesis
12 28	Ferredoxin-NADP reductase	gi|53724876	656	28812	5.78	1.63	Ferredoxin-NADP+ reductase activity
29	Ferritin	gi|53726163	990	18139	5.95	2.25	Oxidative damage protectant Inorganic ion transport and metabolism
**E. More than one function**							
**Secondary metabolites biosynthesis, transport, ATP-binding**							
30	4-Diphosphocytidyl-2-C-methyl-D-erythritol kinase	gi|53724083	804	31931	6.6	4.68	Isopentenyl diphosphate biosynthetic process, Methylerythritol 4-phosphate pathway ,Terpenoid biosynthetic process
31	Fumarate hydratase, class I	gi|126230048	1819	54798	5.99	2.79	Generation of precursor metabolites and energy
32	4-Hydroxy-3-methylbut-2-enyl diphosphate reductase	gi|126227785	1279	35154	5.71	2.05	Isopentenyl diphosphate biosynthetic process, Terpenoid biosynthetic process
**Cellular processes**							
**Cell division**							
33	Trigger factor	gi|53723668	4441	49708	5	2.82	Cell cycle, cell division, protein folding , protein transport

Spot no.	Altered proteins	Accession number	Score	Mass (Da)	pI	Fold change	Biological functions
34	Cell division protein FtsA	gi|53726040	1825	43746	4.96	3.69	FtsZ-dependent cytokinesis
**Transcription-translation process**							
35	DNA gyrase, B subunit	gi|225934904	933	91040	5.8	only P28	DNA-dependent DNA replication<br>DNA topological change
36	RNA methyltransferase	gi|53723479	445	26273	6.08	2.06	RNA binding
37	Serine protein kinase	gi|126228289	718	73430	5.52	1.57	ATP binding<br>Protein kinase activity
38	Elongation factor G	gi|53723857	1561	77397	5.33	only P28	GTPase activity<br>GTP binding<br>Translation elongation factor activity
**Motility**							
39	Flagellar biosynthesis protein	gi|126227917	516	61763	6.09	only P28	Bacterial-type flagellum organization<br>SRP-dependent cotranslational protein targeting to membrane
**Antioxidant**							
41	Putative piperideine-6-carboxylate dehydrogenase	gi|243061628	713	53786	6.35	only P28	Oxidoreductase activity, acting on the aldehyde or oxo group of donors, NAD or NADP as acceptor
**Structure**							
40	Putative porin protein	gi|52212317	781	39155	9.46	1.69	Iron transport
**Virulence factors**							
42	Serine protease	gi|160697961	432	50058	6.99	only P28	Iron binding
43	Carboxy-terminal protease	gi|53724526	769	57006	9.03	only P28	Endoproteases involved in the maturation of proteins destined for the cell envelope.

Spot no.	Altered proteins	Accession number	Score	Mass (Da)	pI	Fold change	Biological functions
44	Transcription accessory protein	gi|53723516	1212	84505	6.13	2.27	Nucleic acid binding, nucleobase-containing compound metabolic process
**Miscellaneous**							
45	Phasin family protein	gi|53723810	1020	19861	5.96	2.20	-
46	Hypothetical protein	gi|126228781	225	30000	5.28	only P28	-
**Down-regulated proteins the 28th passage compared to the first passage**							
**Metabolism**							
**A. Lipid metabolism**							
47	3-Oxoacyl-ACP synthase	gi|53725366	591	43409	5.72	-2.96	Fatty acid biosynthetic process
**B. Energy production and conversion**							
48	Acyl-CoA dehydrogenase	gi|126231768	440	42695	5.86	-9.83	Acyl-CoA dehydrogenase activity,<br>Flavin adenine dinucleotide binding
49	Aldehyde dehydrogenase (NAD) family protein	gi|126226191	1753	50453	5.67	-4.22	Oxidoreductase activity
**C. Amino acid transport and metabolism**							
50	Arginine N-succinyltransferase, beta subunit	gi|157938963	298	37520	5.44	-1.75	Arginine catabolic process
51	Arginine deiminase	gi|53723412	989	46022	5.57	-1.65	Arginine catabolic process to ornithine
**Catabolism**							
52	Phenylacetic acid degradation protein	gi|385373223	2773	60567	5.9	-4.52	Oxidoreductase activity, acting on the aldehyde or oxo group of donors, NAD or NADP as acceptor
53	Catechol 1,2-dioxygenase	gi|52213327	1250	32947	5.62	-3.81	Catechol-containing compound catabolic process

Spot no.	Altered proteins	Accession number	Score	Mass (Da)	pI	Fold change	Biological functions
**Hypothetical protein**							
54<br>55<br>56<br>57	Conserved hypothetical protein	gi|126229704	1290	21654	6.65	-2.04	-
58	Hypothetical protein	gi|52209597	1855	23327	5.14	-4.52	-

Table S3. Summary of altered-proteins of the fifth passage of *B. pseudomallei* reference strain K96243 compared to the first passage in LB broth, incubated 18 h at 37°C in shaking incubator.

Spot no.	Altered proteins	Accession number	Score	Mass (Da)	pI	Fold change	Biological functions
**Up-regulated proteins of the fifth passage compared to the first passage**							
**Cellular processes**							
1	Putative survival protein SurA	gi|157805631	812	48902	7.85	1.62	Chaperone cofactor-dependent protein refolding, Outer membrane assembly, protein stabilization
2	Chaperonin GroL	gi|126226520	3230	57110	5.13	1.73	Protein refolding ATP binding
**Transcription-translation process**							
3	Sua5/YciO/YrdC/YwlC family protein	gi|53725053	92	35533	6.17	1.55	RNA binding
**Structure**							
4	Outer membrane porin	gi|126231279	1846	40142	7.78	4.011	Iron transport
**Down-regulated proteins the fifth passage compared to the first passage**							
**Structure**							
5	Putative lipoprotein	gi|126228333	95	41165	6.15	Absent	Outer membrane protein
**Cellular processes : posttranslational modification, protein turnover, chaperones**							
6<br>7	Peptidyl-prolyl cis-trans isomerase	gi|53723760	1242	17879	5.94	-1.55	Peptidyl-prolyl cis-trans isomerase activity
8	Universal stress family protein	gi|126231980	110	17587	5.95	Absent	Phosphorelay sensor kinase activity
9	GroEL	gi|17940767	101	57110	5.13	Absent	Protein refolding
**Antioxidants**							
10	Ribulose-phosphate3-epimerase	gi|209502968	39	26004	5.97	Absent	Metal iron binding, catalytic activity
11	Organic hydroperoxide resistance protein	gi|126229818	96	14388	6.28	Absent	Response to oxidative stress
12	Superoxide dismutase	gi|53725210	42	18225	6.57	Absent	Superoxide dismutase activity
13	Alkyl hydroperoxide reductase	gi|53723647	1169	18668	6.3	Absent	Peroxidase activity
14	Glyoxalase family protein	gi|126231064	555	14607	6.06	Absent	Glyoxalase activity
**Proteolytic enzymes**							
15<br>16	Putative hydrolase	gi|52209427	116	16294	7.98	Absent	Hydrolase activity

Spot no.	Altered proteins	Accession number	Score	Mass (Da)	pI	Fold change	Biological functions
**Miscellaneous**							
17	Phasin family protein	gi|53723810	83	19861	5.96	-1.8261	-
18<br>19	Conserved hypothetical protein	gi|126231099	60	17996	6.03	-1.8271	-

Table S4. Summary of altered-proteins of the 28th passage of *B. pseudomallei* reference strain K96243 compared to the first passage in LB broth, incubated 18 h at 37°C in shaking incubator.

Spot no.	Altered proteins	Accession number	Score	Mass (Da)	pI	Fold change	Biological functions
**Up-regulated proteins of the 28th passage compared to the first passage**							
**Metabolism**							
**A. Energy production and conversion**							
1<br>2	Phosphoribosylaminoimidazolecarboxamide formyltransferase/IMP cyclohydrolase	gi|169651675	1680	55452	5.97	1.5718	de novo' IMP biosynthetic process<br>IMP cyclohydrolase activity
3	S-adenosylmethionine synthetase	gi|53724461	1013	42615	5.13	1.526	One-carbon metabolic process<br>S-adenosylmethionine biosynthetic process
4	S-adenosyl-L-homocysteine hydrolase	gi|53724307	1933	52168	5.73	1.9857	One-carbon metabolic process<br>S-adenosylhomocysteine catabolic process
5	CTP synthetase	gi|53723786	1895	61003	5.87	2.5138	'de novo' CTP biosynthetic process<br>Glutamine metabolic process
6	ABC transporter	gi|126228033	1389	61618	5.25	1.7217	ATP-binding protein
7<br>8	NADP-dependent malic enzyme/phosphate acetyl/butyryltransferase family protein	gi|126226702	1699	83153	5.81	2.9286	Malate metabolic process
9<br>10	Malate dehydrogenase	gi|217394999	1115	35788	5.91	1.76	Malate metabolic process
11<br>12	Isocitrate dehydrogenase	gi|53725233	1061	46117	5.65	1.7315	Carbohydrate and<br>Malate metabolic process<br>Tricarboxylic acid cycle
13	Succinyl-CoA synthetase subunit beta	gi|53724721	2650	41260	5.25	1.8441	Tricarboxylic acid cycle

Spot no.	Altered proteins	Accession number	Score	Mass (Da)	pI	Fold change	Biological functions
14	Isocitrate lyase	gi|53724111	3012	47745	5.73	3.096	Carboxylic acid metabolic process
15<br>16	Transketolase	gi|126228391	853	74308	5.98	1.762	Iron binding
17<br>18	ATP synthase F0F1 subunit beta	gi|53724016	929	50591	5.26	2.2676	ATP synthesis coupled proton transport
19<br>20	ATP-dependent chaperone ClpB	gi|126227639	1692	95945	5.62	1.9504	Protein metabolic process<br>ATP binding
21	Malic enzyme	gi|53724226	1675	80740	5.92	2.9142	Malate metabolic process
**B. Amino acid transport and metabolism**							
22	N-acetyl-gamma-glutamyl-phosphate reductase	gi|126225763	1498	33704	6.6	1.5599	Arginine biosynthetic process
23	Dihydroxy-acid dehydratase	gi|126226434	1262	58915	5.93	2.4615	Isoleucine biosynthetic process<br>Valine biosynthetic process
24	Glutamine synthetase	gi|53724177	1726	52156	5.26	1.811	Glutamine biosynthetic process
25	1-Deoxy-D-xylulose-5-phosphate synthase	gi|126230272	1348	68238	5.9	1.512	1-Deoxy-D-xylulose 5-phosphate biosynthetic process<br>Terpenoid biosynthetic process<br>Thiamine biosynthetic process
26	Peptide synthase	gi|1025743411	993	37558	5.32	1.8731	Oxidoreductase activity
27	Acetylornithine/succinylornithine aminotransferase	gi|126226136	1617	42865	5.55	2.1756	Arginine biosynthetic process<br>Arginine catabolic process
28	Ornithine carbamoyltransferase	gi|53723413	1598	37993	6.07	7.4351	Arginine biosynthetic process
29	Aspartate-semialdehyde dehydrogenase	gi|126230344	651	39869	5.32	3.034	'de novo' L-methionine biosynthetic process , diaminopimelate biosynthetic process, isoleucine biosynthetic process, lysine

Spot no.	Altered proteins	Accession number	Score	Mass (Da)	pI	Fold change	Biological functions
							biosynthetic process via diaminopimelate, threonine biosynthetic process
30<br>31	Arginine deiminase	gi|53723412	2565	46022	5.57	5.0446	Arginine catabolic process to ornithine
**C. Nucleotide transport and metabolism**							
32	Succinate-CoA ligase, alpha subunit	gi|772973583	1027	30774	6.25	1.8618	Nucleoside triphosphate biosynthetic process<br>Succinate metabolic process<br>Succinyl-CoA metabolic process<br>Tricarboxylic acid cycle
33	Inositol monophosphatase	gi|53723765	1166	29316	5.68	1.7441	Inositol phosphate dephosphorylation
34	Ribose-phosphate pyrophosphokinase	gi|53724084	2162	34189	6.01	1.7492	5-phosphoribose 1-diphosphate biosynthetic process<br>Nucleoside metabolic process<br>Nucleotide biosynthetic process<br>Ribonucleoside monophosphate biosynthetic process
**D. Carbohydrate transport and metabolism**							
35	3-Hydroxybutyrate dehydrogenase	gi|126231356	1307	27448	6.06	2.3838	3-hydroxybutyrate dehydrogenase activity
36	Pyruvate dehydrogenase	gi|126226432	1128	61521	5.49	1.533	Acetyl-CoA biosynthetic process
**E. Inorganic ion transport and metabolism**							
37	Ferredoxin-NADP<br>(H) reductase	gi|53724876	787	28812	5.78	1.5919	Ferredoxin-NADP+ reductase<br>activity

Spot no.	Altered proteins	Accession number	Score	Mass (Da)	pI	Fold change	Biological functions
**Cellular processes**							
38	GroL	gi|126226520	1681	57110	5.13	8.8654	Protein refolding
39	Putative DnaK chaperone protein	gi|52210850	4178	69659	4.94	1.5632	Protein folding
40	GroEL	gi|53725992	3492	57486	5.13	1.7244	Protein refolding
41	Heat shock protein 90	gi|53725892	2948	71105	5.14	2.0762	Unfolded protein binding
42	Universal stress-related protein	gi|126230050	1066	33833	5.46	1.9548	Stress resistance
**Transcription and translation process**							
43	DNA gyrase, B subunit	gi|53724903	921	91060	5.82	2.4347	DNA-dependent DNA replication<br>DNA topological change
44	Putative recombinase A	gi|52208829	856	38149	5.09	2.1526	DNA integration<br>DNA recombination
45	Serine protein kinase	gi|126228289	1724	73430	5.52	2.0875	ATP binding<br>Protein kinase activity
46	Glycyl-tRNA synthetase, beta subunit	gi|126225214	2234	75864	5.89	2.0306	Glycyl-tRNA aminoacylation
47	Phenylalanyl-tRNA synthetase subunit beta	gi|53725301	1259	88565	5.51	2.7758	Phenylalanyl-tRNA aminoacylation
48	Elongation factor Ts	gi|53723738	2315	31173	5.38	1.6197	GTPase activity<br>GTP binding<br>Translation elongation factor activity
49<br>50<br>51	Elongation factor Tu	gi|53723856	2313	42964	5.36	1.5602	GTPase activity , GTP binding ,Translation elongation factor activity
**Antioxidants**							
52	Oxidoreductase	gi|126230666	1442	36542	6.26	1.6035	Oxidoreductase activity
53	Putative epimerase/dehydratase WbiB	gi|3135681	907	39123	5.94	3.0923	Catalytic activity<br>Coenzyme binding
54	Aconitate hydratase	gi|126230436	5205	98484	6.99	2.8615	Aconitate hydratase activity

Spot no.	Altered proteins	Accession number	Score	Mass (Da)	pI	Fold change	Biological functions
**Virulence factor**							
55	Transcription accessory protein	gi|53723516	1649	84505	6.13	2.2721	Nucleic acid binding, nucleobase-containing compound metabolic process
**Down-regulated proteins of the 28th passage compared to the first passage**							
**Metabolism**							
**A. Energy production and conversion**							
56	3-Oxoadipate CoA-succinyl transferase subunit beta	gi|53725280	1381	22273	4.7	- 1.9224	CoA-transferase activity
57	Methylmalonate-semialdehyde dehydrogenase	gi|126229283	874	53899	6.37	Absent	Methylmalonate-semialdehyde dehydrogenase (acylating) activity
58	Putative amino acid ABC transporter	gi|126229515	1330	39897	9.17	- 1.7599	Binding protein
59	Amino acid ABC transporter substrate-binding protein	gi|53724024	361	39477	9.03	Absent	Binding protein
60	Phenylacetic acid degradation protein	gi|126229104	2254	60627	5.73	- 1.7493	Oxidoreductase activity, acting on the aldehyde or oxo group of donors, NAD or NADP as acceptor
61	Putative dTDP-4-keto-6-deoxy-D-glucose 3,5-epimerase	gi|3135676	464	20635	5.43	- 25.0972	dTDP-rhamnose biosynthetic process
62	cmaB protein	gi|126229582	498	35383	5.77	- 9.4926	ATP binding
**B. Lipid metabolism**							
63<br>64	Acetyl-CoA acetyltransferase	gi|52209583	2572	40518	6.62	- 2.7963	Acetyl-CoA C-acetyltransferase activity
65	Ketol-acid reductoisomerase	gi|53725851	493	36245	5.89	Absent	Isoleucine biosynthetic process<br>Valine biosynthetic process
**C. Amino acid transport and metabolism**							
66	Putative protein-L-isoaspartate O-methyltransferase	gi|126225295	1369	24014	5.55	- 2.4094	protein-L-isoaspartate (D-aspartate) O-methyltransferase activity<br>Protein repair

Spot no.	Altered proteins	Accession number	Score	Mass (Da)	pI	Fold change	Biological functions
67	Acetylglutamate kinase	gi|53724381	508	32099	5.06	Absent	Arginine biosynthetic process via ornithine
68	Branched-chain amino acid aminotransferase	gi|53724736	484	33964	6.1	Absent	Isoleucine biosynthetic process<br>Leucine biosynthetic process<br>Valine biosynthetic process
69	3-Isopropylmalate dehydrogenase	gi|126229457	518	38163	5.23	Absent	Leucine biosynthetic process
70	Dihydrodipicolinate synthase	gi|53723773	562	31776	6.26	Absent	Lyase activity
71	2,3,4,5-Tetrahydropyridine-2,6-dicarboxylate N-succinyltransferase	gi|126227913	1005	29519	5.68	Absent	Diaminopimelate biosynthetic process<br>Lysine biosynthetic process via diaminopimelate
**C. Nucleotide transport and metabolism**							
72	Deoxyribose-phosphate aldolase	gi|126231123	1107	26975	5.42	- 1.6235	Deoxyribonucleotide catabolic process<br>Deoxyribose phosphate catabolic process
73<br>74	Acyl-CoA dehydrogenase domain protein	gi|126227018	967	63614	5.52	- 2.1375	Flavin adenine dinucleotide binding<br>Oxidoreductase activity
**D. Carbohydrate transport and metabolism**							
75	Phosphopyruvate hydratase	gi|53723784	1852	45654	4.81	Absent	Glycolytic process
**E. Coenzyme metabolism**							
76	Phosphomethylpyrimidine kinase	gi|225929619	1330	27262	5.84	Absent	Thiamine biosynthetic process
**I. More than one function1; Secondary metabolites biosynthesis, transport , and ATP-binding**							
77<br>78	Acetoacetyl-CoA reductase	gi|238522690	1952	31786	9.29	- 1.993	Secondary metabolites biosynthesis<br>Transport<br>ATP-binding
**Cellular processes**							
79<br>80	Peptidyl-prolyl cis-trans isomerase B	gi|53723760	1299	17879	5.94	Absent	Peptidyl-prolyl cis-trans isomerase activity

Spot no.	Altered proteins	Accession number	Score	Mass (Da)	pI	Fold change	Biological functions
81	Ribokinase	gi|53726105	357	30690	5.45	Absent	Protein refolding
82	Heat shock protein, Hsp20 family	gi|126229259	1288	16016	5.14	-1.8092	Unfolded protein binding
83<br>84	Cell division protein FtsA	gi|53726040	1176	43746	4.96	- 3.8473	Cell division
**Transcription and translation process**							
85	Putative methyltransferase	gi|126232188	337	31155	5.6	- 1.8588	O-methyltransferase activity
86	Transcriptional regulator, LysR family	gi|126226216	380	35944	6.9	Absent	DNA binding
87	Ribosomal subunit interface protein	gi|53724074	320	13656	6.49	Absent	Primary metabolic process
**Structure/cell wall/envelope biogenesis**							
88	LysM domain	gi|126226532	1753	16443	4.54	-1.575	Putative peptidoglycan-binding
89	Putative porin protein	gi|52212317	1940	39155	9.46	- 2.3982	Porin activity<br>Ion transport
90	OmpA family protein	gi|53725212	605	21419	9.72	Absent	Structural molecule activity
**Antioxidants**							
91	Glyoxalase family protein	gi|126231064	652	14607	6.06	-10.9367	Glyoxalase activity
92<br>93	Alkyl hydroperoxide reductase	gi|53723647	1408	18668	6.3	- 1.9813	Peroxidase activity
94	Thioredoxin protein	gi|126226797	898	31063	4.59	- 5.2396	Cell redox homeostasis<br>Glycerol ether metabolic process
**Proteolytic enzymes**							
95	Hydrolase	gi|126231866	912	68135	6.21	Absent	Hydrolase activity
**Miscellaneous**							
96<br>97<br>98<br>99	Conserved hypothetical protein	gi|126228446	647	19871	4.48	-3.182	-
100	Hypothetical protein BMA1518	gi|53723706	667	18325	6.29	-2.668	-
101	Hypothetical protein BMA0944	gi|53725416	613	17434	5.12	-1.6759	-
102	Hypothetical protein BMA3014	gi|53724415	793	18341	5.26	-2.2202	-

Spot no.	Altered proteins	Accession number	Score	Mass (Da)	pI	Fold change	Biological functions
103<br>104	Hypothetical protein BPSL1549	gi|52209597	434	23327	5.14	Absent	-
105<br>106<br>107<br>108<br>109	Phasin family protein	gi|53723810	3774	19861	5.96	Absent	-

Table S5. Primers used in this study.

No.	Primer name	Primer sequence (5'-3')	Product size (bp)	Gene	Sources
1	ArcA F	TCAAGTCGGTGTCCATTC	211	*arcA*	This study
	ArcA R	CAGCAGGTTGTGCATCTC			
2	Ferritin F	ACGCTGTACCTGAAGACG	237	*BPSL2863*	
	Ferritin R	GAGCTGGCGAATCATCT			
3	FlhF F	GACTTCGTTGAGCGTGTC	234	*flhF*	
	FlhF R	ATCTTCGGCAAGATCCTC			
4	Hmp F	GACTTCGTTGAGCGTGTC	234	*hmp*	
	Hmp R	ATCTTCGGCAAGATCCTC			
5	Porin F	ACGTACCAAAGCAACGTC	202	*omp*	
	Porin R	CTTGACGGTTGAACATGC			
6	BPSS0962 F	AGCTCGTCCTTCACCTTC	249	*BPSS0962*	
	BPSS0962 R	GTACGGGTTCGACAACAC			
7	Tuf F	AGTCCACGTGTGCGTAGT	236	*tuf*	
	Tuf R	GAACGTTGGTACGATTGG			
8	GroL F	CTTCAGCTCTTCGACTGC	215	*groL*	
	GroL R	TGAAGGACAAGCTCCAGA			
9	TftC F	CTGTGGGACCGTAACATC	202	*tftC*	
	TftC R	ACCTCGACGATGACCTTC			
10	Stk F	TGTCGGAGGAGATCAAGA	246	*stk*	
	Stk R	CGCGATATTCCTGATACG			
11	Tex F	GGTGTCGAGAAAGTGTCG	192	*tex*	
	Tex R	CTTCACCTTGACGACCTG			
12	GroEL F	TGATCGTCGTGTTTTCCT	215	*groEL*	Vanaporn, M. *et al*. (2008)
	GroEL R	TTCCAAGACCAGTCGACAAC			
13	23s F	TTTCCCGCTTAGATGCTTT	343	23sRNA	Pumirat, P. *et al*. (2010)
	23s R	AAAGGTACTCTGGGGATAA			
